# Implementation of the ABCDEF Bundle for Critically Ill ICU Patients During the COVID-19 Pandemic: A Multi-National 1-Day Point Prevalence Study

**DOI:** 10.3389/fmed.2021.735860

**Published:** 2021-10-28

**Authors:** Keibun Liu, Kensuke Nakamura, Hajime Katsukawa, Peter Nydahl, Eugene Wesley Ely, Sapna R. Kudchadkar, Kunihiko Takahashi, Muhammed Elhadi, Mohan Gurjar, Be Kim Leong, Chi Ryang Chung, Jayachandran Balachandran, Shigeaki Inoue, Alan Kawarai Lefor, Osamu Nishida

**Affiliations:** ^1^Critical Care Research Group, Faculty of Medicine, The Prince Charles Hospital, University of Queensland, Brisbane, QLD, Australia; ^2^Department of Emergency and Critical Care Medicine, Hitachi General Hospital, Hitachi, Japan; ^3^Japanese Society for Early Mobilization, Tokyo, Japan; ^4^Nursing Research, Department of Anesthesiology and Intensive Care Medicine, University Hospital of Schleswig-Holstein, Kiel, Germany; ^5^Critical Illness, Brain Dysfunction, and Survivorship (CIBS) Center, Vanderbilt University School of Medicine, Nashville, TN, United States; ^6^Department of Veterans Affairs Medical Center, Geriatric Research Education and Clinical Center (GRECC), Tennessee Valley Healthcare System, Nashville, TN, United States; ^7^Department of Anesthesiology and Critical Care Medicine, Johns Hopkins University School of Medicine, Baltimore, MD, United States; ^8^Department of Physical Medicine and Rehabilitation, Johns Hopkins University School of Medicine, Baltimore, MD, United States; ^9^Department of Pediatrics, Johns Hopkins University School of Medicine, Baltimore, MD, United States; ^10^Department of Biostatistics, M&D Data Science Center, Tokyo Medical and Dental University, Tokyo, Japan; ^11^Faculty of Medicine, University of Tripoli, Tripoli, Libya; ^12^Department of Critical Care Medicine, Sanjay Gandhi Post Graduate Institute of Medical Sciences (SGPGIMS), Lucknow, India; ^13^Department of Rehabilitation Medicine, Sarawak General Hospital, Kuching, Malaysia; ^14^Department of Critical Care Medicine, Samsung Medical Center, Sungkyunkwan University School of Medicine, Seoul, South Korea; ^15^Rehabilitation Department, Woodlands Health Campus, Yishun, Singapore; ^16^Emergency and Critical Care Center, Kobe University Hospital, Kobe, Japan; ^17^Department of Disaster and Emergency Medicine, Graduate School of Medicine, Kobe University, Kobe, Japan; ^18^Department of Surgery, Jichi Medical University, Tochigi, Japan; ^19^Department of Anesthesiology and Critical Care Medicine, Fujita Health University School of Medicine, Toyoake, Japan

**Keywords:** ABCDEF bundle, COVID-19, ICU diary, ICU liberation bundle, pandemic (COVID-19)

## Abstract

**Background:** Data regarding delivery of evidence-based care to critically ill patients in Intensive Care Units (ICU) during the COVID-19 pandemic is crucial but lacking. This study aimed to evaluate the implementation rate of the ABCDEF bundle, which is a collection of six evidence-based ICU care initiatives which are strongly recommended to be incorporated into clinical practice, and ICU diaries for patients with and without COVID-19 infections in ICUs, and to analyze the impact of COVID-19 on implementation of each element of the bundle and independent associated factors.

**Methods:** A world-wide 1-day point prevalence study investigated the delivery of the ABCDEF bundle and ICU diary to patients without or with COVID-19 infections on 27 January 2021 *via* an online questionnaire. Multivariable logistic regression analysis with adjustment for patient demographics evaluated the impact of COVID-19 and identified factors in ICU administrative structures and policies independently associated with delivery.

**Results:** From 54 countries and 135 ICUs, 1,229 patients were eligible, and 607 (49%) had COVID-19 infections. Implementation rates were: entire bundle (without COVID-19: 0% and with COVID-19: 1%), Element A (regular pain assessment: 64 and 55%), Element B (both spontaneous awakening and breathing trials: 17 and 10%), Element C (regular sedation assessment: 45 and 61%), Element D (regular delirium assessment: 39 and 35%), Element E (exercise: 22 and 25%), Element F (family engagement/empowerment: 16 and 30%), and ICU diary (17 and 21%). The presence of COVID-19 was not associated with failure to implement individual elements. Independently associated factors for each element in common between the two groups included presence of a specific written protocol, application of a target/goal, and tele-ICU management. A lower income status country and a 3:1 nurse-patient ratio were significantly associated with non-implementation of elements A, C, and D, while a lower income status country was also associated with implementation of element F.

**Conclusions:** Regardless of COVID-19 infection status, implementation rates for the ABCDEF bundle, for each element individually and an ICU diary were extremely low for patients without and with COVID-19 infections during the pandemic. Strategies to facilitate implementation of and adherence to the complete ABCDEF bundle should be optimized and addressed based on unit-specific barriers and facilitators.

## Introduction

For patients in the intensive care unit (ICU), evidence-based treatment such as the ABCDEF bundle (1–4) and ICU diary (5), should be established as part of routine clinical practice because they are strongly linked not only to short-term outcomes of ICU patients (6, 7) but also their long-term function and quality of life (QOL) (8). Recent studies confirmed that the beneficial effects of the ABCDEF bundle are maximized when provided as a combination of elements or as the entire bundle (9, 10).

However, drastic changes in practice related to the COVID-19 pandemic, including unbalanced resources, overwhelmed facility capacity, and strict infectious regulations, occurred world-wide and prevented ICU staff from performing evidence-based approaches to patient care in ICUs (11). Our recent survey demonstrated low implementation rates of each element and the entire ABCDEDF bundle and other supportive ICU care for patients with COVID-19 infections in the ICU (12). We could not assess the total impact of COVID-19 in the ICU because of a lack of data on patients without COVID-19 infections. To overcome low implementation rates which result in poor outcomes of ICU patients (13), a number of studies proposed efficient ways associated with the ICU administrative structure and environment to promote evidence-based ICU care before the pandemic (14–16). Nonetheless, clinical data on promoting factors and barriers during the pandemic are lacking and these factors could vary when treating patients without or with COVID-19 infections because the policy of less physical contact in a short time while wearing protective personal equipment was generally enforced only for patients with COVID-19 infections. Moreover, low- and middle-income countries are more vulnerable to these resource-dependent changes (17).

Therefore, we conducted a 1-day point prevalence study, to investigate the implementation rate of evidence-based ICU care for both patients without and with COVID-19 infections and the impact of COVID-19 infections on implementation on a world-wide scale to capture the current clinical practice situation. We sought to identify ICU-related factors associated with implementation in the ICU.

## Materials and Methods

### Study Design and Settings

This was an international 1-day point prevalence study conducted on 27 January 2021, with approval by the ethics committee of the Saiseikai Utsunomiya Hospital (2020-69) and pre-registration in UMIN (ID: 000040405). The study design and construction followed the STROBE cross-sectional guidelines.

The study committee recruited participants from January 8 to 26 by disseminating an invitation letter to members of the Indian Society of Critical Care Medicine, the Korean Society of Critical Care Medicine, and other local or national networks in collaboration with regional/national coordinators (Appendix 1). The invitation letter included a brief introduction of this study, a specific link to the web site explaining the study details (https://form.jsea2005.org/isiic-II-study/), ethical considerations, and the URL for registration created by Google Forms (Google Inc.). According to the Ethical Guidelines for Medical and Health Research Involving Human Subjects in Japan (18), ethical approval at each participating institution was waived because of the anonymous nature of this study which will not collect specific data that could identify ICUs or individual patients. All ICUs which agreed to the study policies could register and there were no exclusion criteria. The name of one representative for each participating ICU, the name of the hospital, and its country were registered to confirm the reliability of data sources (Appendix 1).

### Study Process

The study committee requested all registered representatives to provide background data for their hospitals and ICUs *via* a Google Form starting 20 January 2021, before the survey date. The questionnaire used to obtain background data (e.g., number of hospital beds, ICU beds, COVID-19 specific ICU beds, nurse-to-patient ratio) is shown in Appendix 2 (18 questions, 3 min). The income level was classified according to the World Bank Country Classification (https://datahelpdesk.worldbank.org/knowledgebase/articles/906519-world-bank-country-and-lending-groups) according to the country where the participating ICUs are located, which was obtained as the background data. Each representative received a different Facility Registration Number automatically soon after the completion of the questionnaire. On the survey date, 27 January 2021, the URL for the survey of evidence-based and supportive ICU care (21 questions, 3–5 min, Google Form) were sent to all registered representatives. All representatives were asked to input the institution-specific Facility Registration Number at the first question, and only those who had it could continue to complete the survey (Appendix 3). The questions in the survey were pre-reviewed by the study co-authors and pre-tested by collaborative physicians and nurses listed in the acknowledgment. The URL for the survey was open from January 27 to 30.

### Data Collection

In the survey, patient demographics, such as age, gender, Body Mass Index (BMI), and ICU length of stay as of the survey date, use of medical devices, continuous use of neuromuscular blockade, vasoactive, analgesia, and sedation agents, prone positioning and its duration, the presence of a target/goal of each ICU care modality given to ICU patients on the survey date, and the implementation of each element of the ABCDEF bundle, ICU diary provided on the survey data were collected. The operational definitions of each element of the ABCDEF bundle and ICU diary (Table 1) were provided to respondents at the appropriate place in the survey. The representatives completed one questionnaire for each patient, except for patients who were terminally ill and receiving palliative care. For example, if there were three ICU patients in the ICU, the representative needed to complete the questionnaire for the survey of evidence-based and supportive ICU care three times. Data obtained from the survey were anonymous both for patients and institutions, the data of evidence-based and supportive ICU care was linked to data of the background data for their hospitals and ICUs by the facility-specific Facility Registration Number.

**Table 1:** Operational definitions of evidence-based and supportive ICU care.

**Elements of the ABCDEF bundle**	**Operational definition**
Element A^a^^,^^b^	Regular standardized **PAIN** assessment using valid and reliable pain assessment scales six times or more per day. The pain assessment scales include Numerical Rating Scale (NRS), Critical-care Pain Observation Tool (CPOT), Behavioral Pain Scale (BPS), and others.
Element B^a^^,^^b^	Both **SPONTANEOUS AWAKENING TRIALS** and **SPONTANEOUS BREATHING TRIALS**. The spontaneous awakening trial is cessation of sedatives and narcotics or similar protocols to evaluate consciousness. The spontaneous breathing trial is to turn the respiratory rate to zero with eight or less of pressure support ventilation or similar local protocol to evaluate whether the patient meets the requirements for extubation.
Element C^a^^,^^b^	Regular standardized **SEDATION** assessment using valid and reliable sedation assessment scales (※4) six times or more per day. The sedation assessment scales include Richmond Agitation- Sedation Scale (RASS), Sedation-Agitation Scale (SAS), Ramsay Sedation Scale, and others.
Element D^a^^,^^b^	Regular standardized **DELIRIUM** assessment using valid and reliable delirium monitoring tools (※5) two times or more per day. The delirium assessment tools include Confusion Assessment Method for ICU (CAM-ICU), Intensive Care Delirium Screening Checklist (ICDSC), and others.
Element E^a^^,^^b^^,^^c^	**MOBILITY** activities that were out of bed or higher. It is equal to a score of 4 or higher according to the Intensive Care Unit Mobility Scale^*c*^ (i.e., dangling at edge of bed, standing at side of bed, walking to bedside chair, marching in place, walking in room or hall.).
Element F^a^^,^^b^	**FAMILY ENGAGEMENT AND EMPOWERMENT** that a family member/significant other of this patient is educated regarding the ABCDEF bundle and/or participates in at least one of the following: rounds; conference; plan of care; or ABCDEF bundle related care, e.g., re-orientation, calming talks etc. This element could be conducted in person or online.
**Other ICU care**	
ICU diary^d^	An **ICU DIARY** is a patient journal, written by staff and families for several purposes, and includes daily entries about what happened.

*ICU intensive care unit. These definitions are based on the following references*.

a *Pun et al. (13)*.

b *Liu et al. (12)*.

c *Hodgson et al. (19). IMS, 0: Nothing (lying in bed, passive exercise), 1: sitting in bed, exercises in bed; 2: passively moved to chair (no standing); 3: sitting over edge of bed; 4: standing; 5: transferring bed to chair; 6: marching in place (at bedside); 7: walking with assistance of two or more people; 8: walking with assistance of one person; 9: walking independently with a gait aid; 10: walking independently without a gait aid*.

d *Nydahl and Deffner (5)*.

All the data were stored online (Google Drive, Google Inc.) and managed or exported by the authorized person out of the authors (Appendix 1).

### Outcomes

The primary outcome was the implementation rate of the entire ABCDEF bundle. Secondary outcomes were the implementation rates for each element of the ABCDEF bundle, including element A (regular pain assessment), element B [both spontaneous awakening trials (SAT) and spontaneous breathing trials (SBT)], element C (regular sedation assessment), element D (regular delirium assessment), element E (early mobility and exercise), and element F (family engagement and empowerment), and an ICU diary.

The implementation of element E during mechanical ventilation, the implementation of element F performed online, and visitation policies for family members were also described. Independent factors associated with successful implementation of each element of the ABCDEF bundle were evaluated by multivariable logistic regression analysis.

### Statistical Analysis

Non-normally distributed continuous data were reported as medians with interquartile range (IQR). Categorical data were described as numbers or percentages. Comparisons of patient demographics, implementation of the ABCDEF bundle, and the ICU diary between the groups of patients with out and with COVID-19 infections were made with the Mann-Whitney *U*-test for non-normally distributed continuous data and the chi-squared test and Fisher's exact test for categorical data appropriately. There was no missing data.

In multivariable logistic regression analysis with adjustment for patient demographics, the association between the implementation of each element of the ABCDEF bundle and the presence of COVID-19 infection or ICU administrative structures was investigated. Patient demographics included length of ICU stay, age, gender, body mass index, use of mechanical ventilation, extracorporeal membrane oxygenation including veno-venous and veno-arterial, renal replacement therapy, and left ventricular unloading device, continuous use of neuromuscular blockade, vasoactive drugs, analgesia agents and sedation agents, and prone positioning. The following variables were changed to factors and used in the multivariable logistic regression analysis: number of hospital beds, nurse-to-patient ratio, frequency of multidisciplinary rounds, number of visiting hours for a family, type of hospital and ICU, primary responsibility to make decisions to implement the ABCDEF bundle, age, body mass index, income level. As a sub-analysis, associated independent factors among ICU administrative structures for each group (non-COVID-19 and COVID-19) were evaluated through the stepwise method with Akaike information criterion and with adjustments of the same variables of patients demographics described above. The stepwise method was used to focus on significant factors. In the sub-analysis, the variables that the number of patients allocated to the category is too few (≤5 patients) to create a suitable model were excluded from multivariable logistic regression analysis.

The calculated sample size with 95% power and a two-sided alpha of 0.05 was 508 patients under the assumption of the implementation rate of the entire ABCDEF bundle for patients without and with COVID-19 infections (8 and 1%, respectively) based on previous surveys (12, 13). All statistical analyses were carried out using EZR (Saitama Medical Center, Jichi Medical University, Saitama, Japan) (20) and R (R Project, Vienna, Austria). The *p*-value was reported as two-sided and *p* <0.05 was considered statistically significant.

## Results

### Background of Hospitals and ICUs

**Figure 1:**
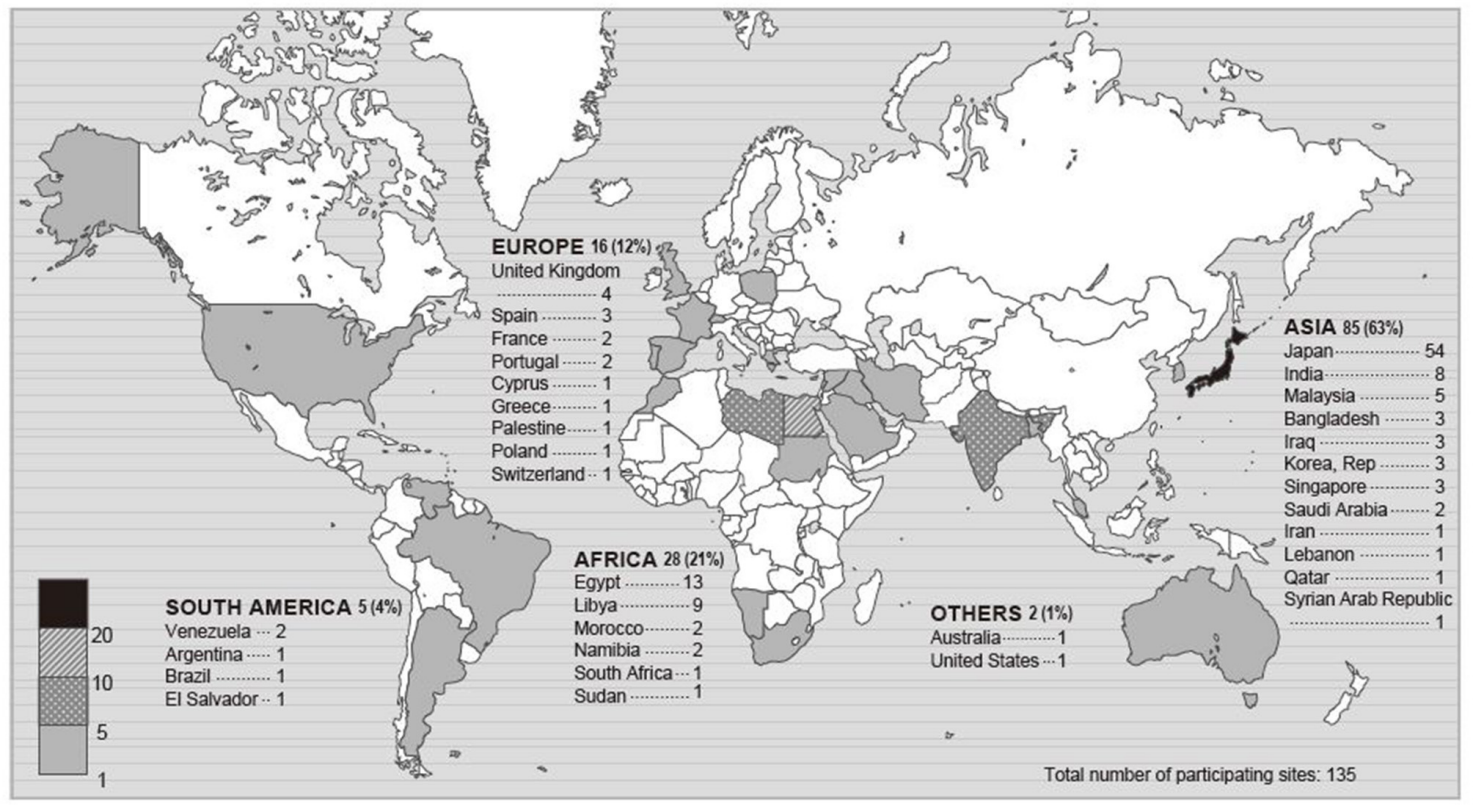
Map of participating sites. The numbers in this figure indicate the number of participating ICUs in each country or a percentage. Percentage indicates the proportion of the total participating ICUs.

**Figure 2:**
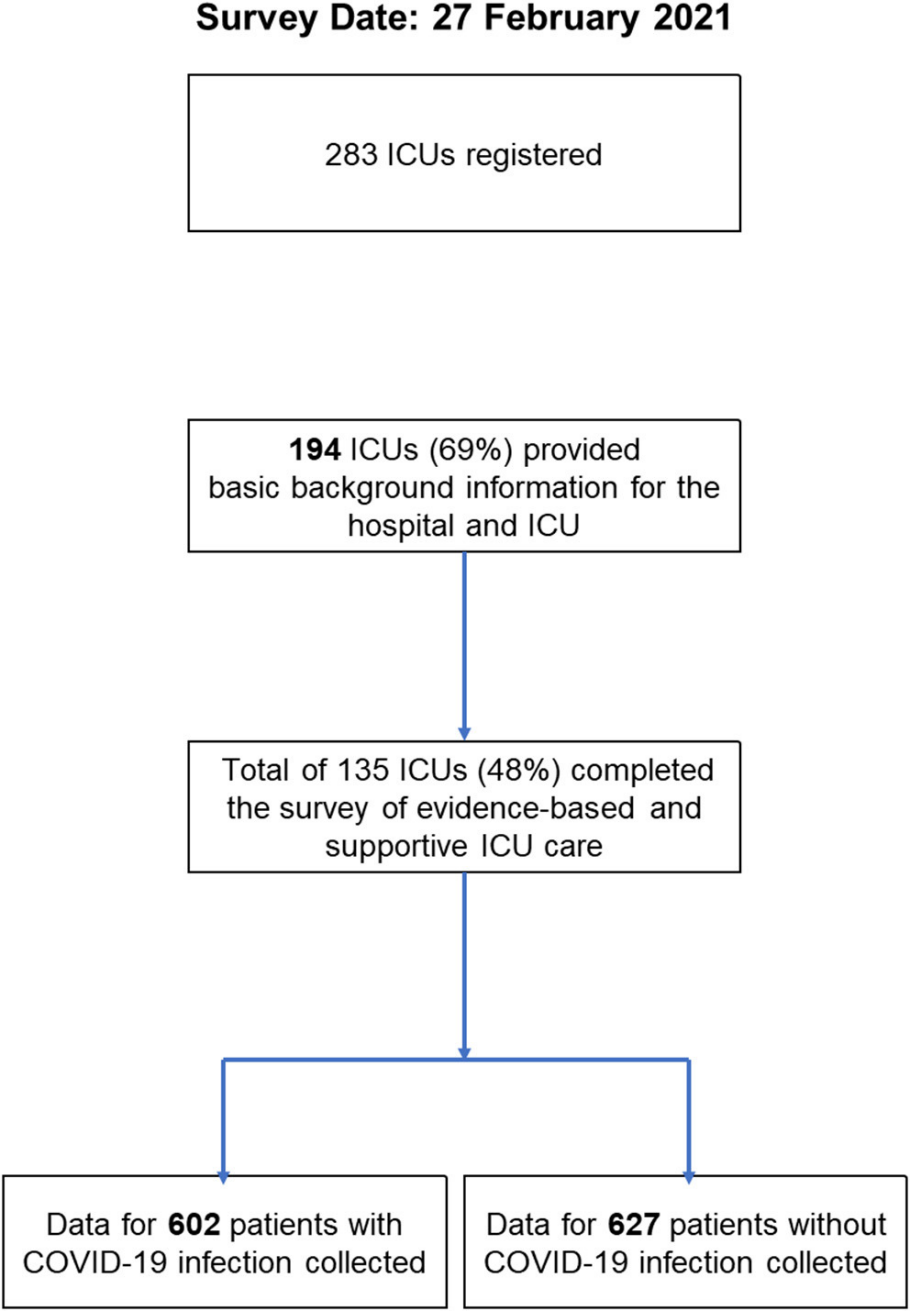
Study flow chart.

Of 283 registered ICUs, 135 ICUs completed the survey (response rate 48%) (Figures 1, 2). Respondents included 78% physicians. The most common size of participating hospitals was 800 beds or more, with a median of 14 ICU beds and 4 beds allocated for patients with COVID-19 infections (Table 2). The nurse: patient ratio was 1:2 in 53% of ICUs. Multidisciplinary rounds were conducted significantly less frequently for patients with COVID-19 infections (*p* = 0.004). Compared to before the pandemic, family visiting hours to patients both without and with COVID-19 infection were reduced (<0.001 and *p* = 0.004, respectively), and more stringent restrictions imposed on families of patients with COVID-19 infections (*p* <0.001). A specific protocol for each element of the ABCDEF bundle was in place in <50% of ICUs except for a protocol for pain management (51%).

**Table 2:** Background, administrative structure, and policies of participating hospitals and ICUs.

**Characteristic**	**Participating ICUs** **(n = 135)**
Number of hospital beds (beds), *n* (%)	
x <200	21 (16%)
200 ≤ x <400	19 (14%)
400 ≤ x <600	27 (20%)
600 ≤ x <800	26 (19%)
x ≥ 800	42 (31%)
Total number of ICU beds (beds), median [IRQ]	14 [10–25]
ICU beds exclusively for patients with COVID-19 infection (beds), median [IRQ]^a^	4 [2–10]
Number of participating ICUs where tele-medicine is available	6 (4%)
Nurse-to-patient ratio, *n* (%):	
1	41 (30%)
2	72 (53%)
x ≥ 3	21 (16%)
Number of participating ICUs which belong to the following income level, *n* (%)^b^	
Low and lower middle-income countries	30 (22%)
Upper middle-income countries	26 (19%)
High income countries	79 (59%)
The frequency of multidisciplinary rounds for patients **WITH** COVID-19 in the ICU, *n* (%)	
Not applicable	35 (26%)
Daily	78 (58%)
Other frequency (at least once a week or a month)	22 (16%)
The frequency of multidisciplinary rounds for patients **WITHOUT** COVID-19 in the ICU, *n* (%)	
Not applicable	14 (10%)
Daily	91 (67%)
Other frequency (at least once a week or a month)	28 (21%)
Number of visiting hours in the ICU for a family per day **BEFORE** the COVID-19 pandemic (hours), *n* (%)	
No visiting hours available	9 (7%)
0 < x <6	87 (64%)
6 ≤ x ≤ 24	39 (29%)
Number of visiting hours to a patient **WITHOUT** COVID-19 infection per day in the ICU **AFTER** the COVID-19 pandemic (hours), *n* (%)	
No visiting hours available	66 (49%)
0 < x <6	64 (47%)
6 ≤ x ≤ 24	5 (4%)
Number of visiting hours to a patient **WITH** COVID-19 infection per day in the ICU **AFTER** the COVID-19 pandemic (hours), *n* (%)	
No visiting hours available	106 (79%)
0 < x <6	26 (19%)
6 ≤ x ≤ 24	3 (2%)
Presence of a specific written protocol for each element of the ABCDEF bundle, *n* (%)	
Element A: Pain management	69 (51%)
Element B: Spontaneous Awakening Trial (SAT) management	47 (35%)
Element B: Spontaneous breathing trial (SBT) management	64 (47%)
Element C: Sedation management	66 (49%)
Element D; Delirium management	54 (40%)
Element E: Early mobility and exercise	61 (45%)
Element F: Family engagement and empowerment	13 (10%)
No written protocol associated with the ABCDEF bundle	18 (13%)

*Data in table are presented as number (%) or median [Interquartile range]. ICU, intensive care unit; IQR, interquartile range*.

a *Among 135 participating ICUs, 106 (79%) ICUs accommodated the ICU beds exclusively allocated for patients with COVID-19 infection*.

b *The income level was classified according to the World Bank Country and Lending Group. Available online at: https://datahelpdesk.worldbank.org/knowledgebase/articles/906519-world-bank-country-and-lending-groups (accessed 4 March in 2021)*.

The details of the types of hospitals and ICUs participating, professionals dedicated to the ICU, and the personnel with primary responsibility for implementing the ABCDEF bundle are shown in Supplementary Table 1.

### Patient Demographics

There were significant differences in the demographics of the two groups for ICU length of stay, age, BMI, gender, use of mechanical ventilation (49 vs. 66%) and left-ventricular unloading device, continuous use of neuromuscular blockade, analgesia and sedation agents, prone positioning, and its duration (Table 3). The two groups were not significantly different regarding the use of extracorporeal membrane oxygenation and renal replacement therapy and continuous use of vasoactive drugs (Table 3). The target/goal for pain control was less frequently applied to patients with COVID-19 infections and more sedation was given to them, while no difference was seen for early mobilization and rehabilitation.

**Table 3:** Comparison of demographics of patients without and with COVID-19 infections.

**Variable**	**Patients without COVID-19 infection (*n* = 627)**	**Patients with COVID-19 infection (*n* = 602)**	***P*-value**
ICU length of stay (days), median [IQR]	5 [2–10]	9 [2–10]	<0.001
Age (years), *n* (%)			<0.001
x < 50	190 (30%)	107 (18%)	
50 ≤ x <60	90 (13%)	132 (22%)	
60 ≤ x <70	120 (19%)	193 (32%)	
x ≥ 70	227 (36%)	170 (28%)	
Gender (male), *n* (%)	391 (62%)	425 (70%)	0.003
Body mass index (kg/m^2^), *n* (%)			<0.001
x < 18.5	84 (13%)	10 (2%)	
18.5 ≤ x <25	310 (49%)	150 (25%)	
25 ≤ x <30	155 (25%)	218 (36%)	
x ≥ 30	78 (12%)	224 (37%)	
Use of medical devices, *n* (%)			
Mechanical ventilation	306 (49%)	395 (66%)	<0.001
Extracorporeal membrane oxygenation^a^	18 (3%)	30 (5%)	0.076
Renal replacement therapy	66 (11%)	56 (9%)	0.505
Left ventricular unloading device (Impella®, IABP)	10 (2%)	1 (0%)	0.012
Patients receiving continuous use of neuromuscular blockade, *n* (%)	19 (3%)	159 (26%)	<0.001
Patients receiving continuous use of vasoactive drugs, *n* (%)	208 (33%)	186 (31%)	0.427
Patients receiving continuous use of analgesia agents, *n* (%)	291 (46%)	358 (59%)	<0.001
Patients receiving continuous use of sedation agents, *n* (%)	233 (37%)	356 (59%)	<0.001
Patients receiving prone positioning, *n* (%)	17 (3%)	209 (34%)	<0.001
Scheduled total number of hours of prone positioning (hours), *n* (%)			<0.001
0 hours (no performing)	191 (30%)	333 (55%)	
0 < x < 6	11 (2%)	58 (10%)	
6 ≤ x < 12	2 (0%)	38 (6%)	
12 ≤ x < 18	4 (1%)	57 (9%)	
18 ≤ x ≤ 24	0 (0%)	56 (9%)	
Not candidate (i.e., because of no respiratory failure)	419 (67%)	60 (10%)	
Presence of a target or goal applied to ICU patients on the survey date, *n* (%)			
Pain	255 (41%)	201 (33%)	0.009
Sedation	280 (45%)	393 (65%)	<0.001
Mobilization/Rehabilitation	296 (47%)	261 (43%)	0.187

*Data in table are presented as median [Interquartile range] or number (%). ICU, intensive care unit; IQR, interquartile range; IABP, intra-aortic balloon pump*.

a *Among the 18 patients received extracorporeal membrane oxygenation WITHOUT COVID-19 infection 9 were Veno-Venous extracorporeal membrane oxygenation and 9 were Veno-Arterial extracorporeal membrane oxygenation. Among the 30 patients received extracorporeal membrane oxygenation WITH COVID-19 infection, 29 were Veno-Venous extracorporeal membrane oxygenation and 9 were Veno-Arterial extracorporeal membrane oxygenation*.

### Implementation of Evidence-Based ICU Care

The implementation of the entire ABCDEF bundle, including elements A, B, C, D, E, and F which targets patients undergoing mechanical ventilation and continuous sedation, was rarely performed for patients both without and with COVID-19 infections (without COVID-19: 0% vs. with COVID-19: 1%, *p* = 0.53) (Table 4). The rate was similar if one element of the six was excluded (2 vs. 3%, *p* = 0.59). Given elements A, C, D, E, and F which target all ICU patients, the implementation rate of all of these elements was low (1 vs. 3%. *P* = 0.07), even when one of the five was excluded (5 vs. 7%, *p* = 0.08).

**Table 4:** Comparison of implementation of evidence-based and supportive ICU care.

**Variables**	**Total ICU patients (*n* = 1,229)**	**The patients without COVID-19 infection (*n* = 627)**	**The patients with COVID-19 infection (*n* = 602)**	***P*-value**
**Primary outcomes: implementation of an entire or a synchronized form of the ABCDEF bundle**				
Performing an entire of the ABCDEF bundle, *n* (%)^a^	2 (0%)	0 (0%)	2 (1%)	0.241
Performing any combinations of five of six elements: A, B, C, D, E, and F, *n* (%)^a^	15 (3%)	4 (2%)	11 (3%)	0.070
Performing an entire of the ABCDEF bundle except B, *n* (%)^b^	25 (2%)	8 (1%)	17 (3%)	0.068
Performing any combinations of four of five elements: A, C, D, E, and F, *n* (%)^b^	76 (6%)	31 (5%)	45 (7%)	0.075
**Secondary outcomes: implementation of each element in the ABCDEF bundle**				
Element **A**, *n* (%)	731 (59%)	400 (64%)	331 (55%)	0.002
Element **B:** both SAT and SBT^a^	67 (12%)	33 (17%)	34 (10%)	0.030
**SAT** under continuous sedation, *n* (%)^c^	98 (17%)	49 (21%)	49 (14%)	0.024
**SBT** during mechanical ventilation, *n* (%)^d^	154 (22%)	90 (29%)	64 (16%)	<0.001
Element **C**, *n* (%)	650 (53%)	283 (45%)	367 (61%)	<0.001
Element **D**, *n* (%)	452 (37%)	244 (39%)	208 (35%)	0.124
Element **E**, *n* (%)	175 (14%)	77 (12%)	98 (16%)	0.050
Element **E** during mechanical ventilation, *n* (%)^d^	44 (6%)	19 (6%)	25 (6%)	1
Element **F**, *n* (%)	279 (23%)	98 (16%)	181 (30%)	<0.001
Element **F** which was conducted *via* online, *n* (%)	150 (12%)	26 (4%)	124 (21%)	<0.001
Visiting arrangement for a family to meet patients in the ICU, *n* (%)				
Meeting not allowed	630 (51%)	297 (47%)	333 (55%)	0.006
In person	307 (25%)	251 (40%)	56 (9%)	<0.001
Visiting through the glass outside the room	36 (3%)	12 (2%)	24 (4%)	0.040
Using electronic device (using a monitor such as phone/video)	269 (24%)	75 (12%)	194 (32%)	<0.001
**Implementation of other evidence-based and supportive ICU cares**				
ICU Diary, *n* (%)	234 (19%)	106 (17%)	128 (21%)	0.059

*Data in table are presented as number (%) or median [Interquartile range]. ICU, intensive care unit; SAT, spontaneous awakening trials; SBT, spontaneous breathing trials; IQR, interquartile range*.

a *The targeted ICU patients are those who receive continuous sedation and mechanical ventilation at the same time. A total number of those patients are 539, including 340 patients with COVID-19 infection and 199 patients without COVID-19 infection. Percentages were calculated by dividing by these numbers of sedated and ventilated patients*.

b *The targeted ICU patients are all ICU patients on the survey date*.

c *The targeted ICU patients are those who receive continuous sedation. A total number of those patients are 589, including 356 patients with COVID-19 infection and 233 patients without COVID-19 infection. Percentages were calculated by dividing by these numbers of sedated patients*.

d *The targeted ICU patients are those who receive mechanical ventilation. A total number of those patients are 701, including 395 patients with COVID-19 infection and 306 patients without COVID-19 infection. Percentages were calculated by dividing by these numbers of ventilated patients*.

Element A (64 vs. 55%), element B (17 vs. 10%), SAT (21 vs. 14%), and SBT (29vs. 16%) were implemented significantly less often for patients with COVID-19 infection, while element C (45 vs. 61%), element F (16 vs. 30%) and the online conduct of element F (4 vs. 21%), were performed significantly more frequently for patients with COVID-19 infections. There was no significant difference in the implementation of element D (39 vs. 35%), element E (22 vs. 25%), even while patients were undergoing mechanical ventilation (6 vs. 6%), and the ICU diary (17 vs. 21%). In-person visits were significantly less frequently allowed but online visits using electronic devices were more often used for the families of patients with COVID-19 infection.

### Independent Factors Associated With Implementation of the ABCDEF Bundle

**Figure 3:**
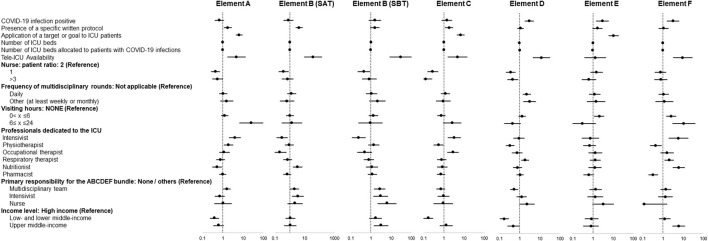
Independent factors associated with implementation of each element of the ABCDEF bundle. Data in figure are shown as adjusted odds ratio with 95% confidential interval.

In multivariable regression analysis adjusted for baseline conditions, the presence of COVID-19 infection was not associated with non-implementation of individual elements of the bundle, but was significantly associated with implementation of elements D, E, and F (Figure 3).

Among ICU administrative structural elements, specific factors associated with implementation were identified for each element of the bundle. Element A: presence of a written protocol and a target/goal, management as a tele-ICU, the presence of dedicated intensivists, and responsibility by a multidisciplinary team. Element B (SAT): presence of a written protocol, management as a tele-ICU, and responsibility by a multidisciplinary team and intensivists. Element B (SBT): management as a tele-ICU, responsibility by a multidisciplinary team, intensivists, and nurses, and being in an upper-middle-income country. Element C: presence of a written protocol and a target/goal, management as a tele-ICU, and presence of dedicated intensivists. Element D: management as a tele-ICU, performing multidisciplinary rounds daily and at least once a week or month, presence of dedicated respiratory therapists, and responsibility by nurses. Element E: presence of a target/goal and visiting hours (0 < x < 6 h). Element F: management as a tele-ICU, visiting hours (0 < x ≤ 24 h), presence of dedicated Intensivists, respiratory therapist, and nutritionist, and being in an upper-middle-income country.

**Figure 4:**
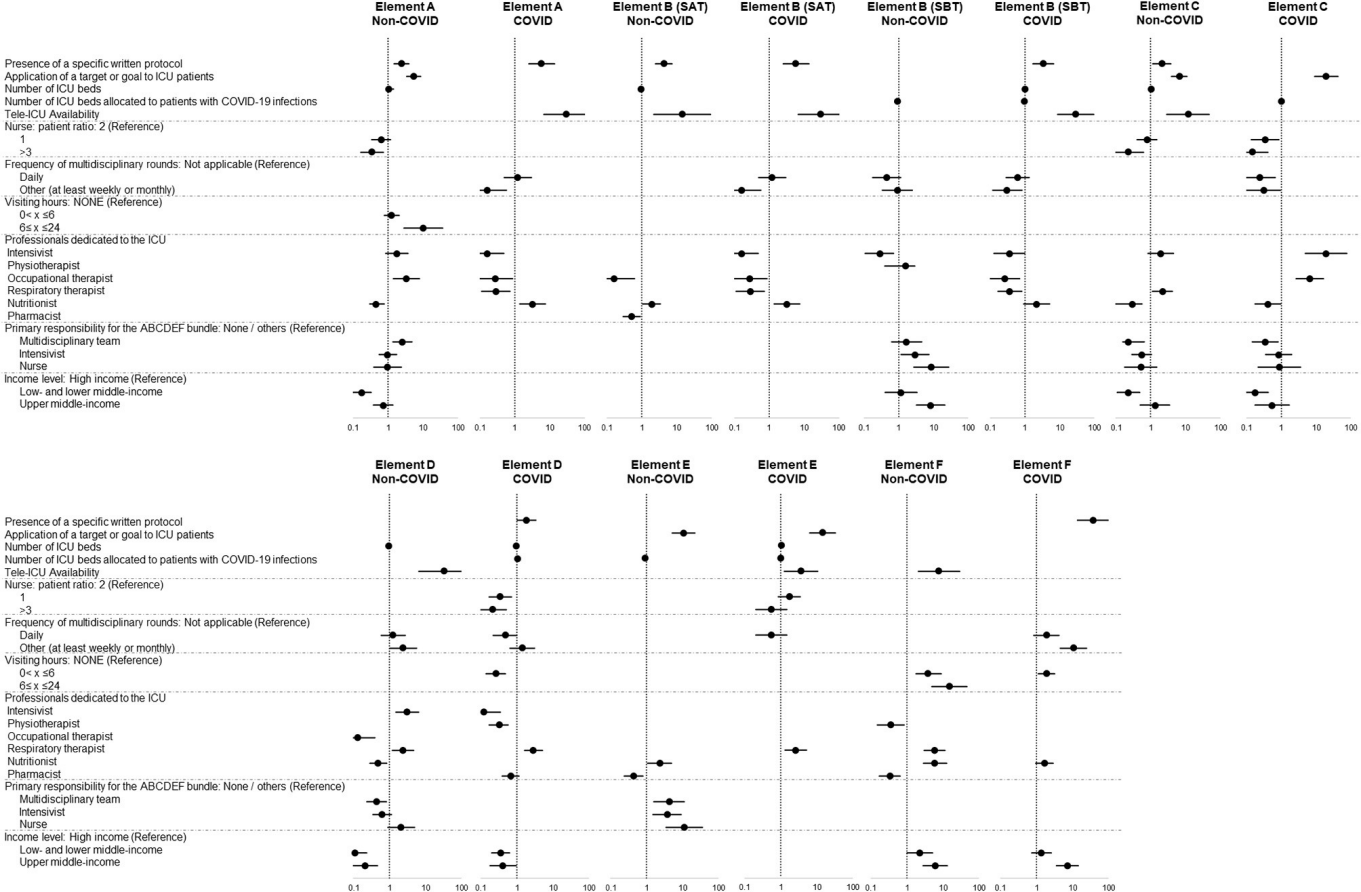
Differences in associated independent factors in patients without and with COVID-19 infections. Data in figure are shown as adjusted odds ratio with 95% confidential interval. The blank lines in the figure are the variables which are excluded from the multivariable logistic regression analysis because the number of patients allocated to the category is too small (≤5 patients) to create the suitable model or the variables which are removed through the stepwise method with Akaike information criterion.

In the sub-analysis, a variety of different independent factors were identified for patients without and with COVID-19 infections (Figure 4). The presence of a specific written protocol, application of a target/goal, and tele-ICU management were associated with implementation of elements of the bundle in both groups. For patients without and with COVID-19 infections, a 1:1 nurse-patient ratio and daily multidisciplinary round were not significant independent factors, and being in lower- and lower-middle-income countries and a 3:1 nurse-patient ratio were significantly associated with a lower rate of implementation of elements C and D for both groups and element A for those without COVID-19 infections.

## Discussion

This world-wide 1-day prevalence study demonstrates that implementation of the entire ABCDEF bundle, or its individual elements and an ICU diary for patients without and with COVID-19 infections is extremely low even though the implementation rate of specific individual elements of the ABCDEF bundle was different for the two groups. The presence of COVID-19 infection was not a factor preventing implementation. A variety of ICU-related factors were identified as independently associated facilitators or barriers for the implementation of the ABCDEF bundle, and these were different for each element, comparing patients without and with COVID-19 infections.

Implementation of the ABCDEF bundle is much lower in this study compared with that reported by a survey before the pandemic (13, 21), but similar to a survey conducted in the early stages of the COVID-19 pandemic on 3 June and 1 July 2020 (12) (Table 5). These results suggest that COVID-19 affects the care of not only patients with COVID-19 infections but also patients without COVID-19 infections and this effect may have been present since the beginning of the pandemic. Numerous studies strongly show that each element of the ABCDEF bundle or an ICU diary itself has a beneficial effect on patient outcomes (7, 22–26), while low and incomplete implementation can result in adverse outcomes including increased time of ventilatory support, longer ICU and hospital lengths of stay, increased incidence of delirium, functional disability, and increased medical costs and mortality (13, 27). Efficient ways to incorporate evidence-based ICU care into clinical practice during the pandemic are urgently needed. To note, the studies included in Table 5 had various methods to collect data, hence the simple comparison might lead to the misleading. Further and continuous international surveys with same methodology are necessary to follow the implementation rate of the ABCDEF bundle in future. In addition, studies to investigate the effects of the ABCDEF bundle and other evidence-based ICU care are expected.

**Table 5:** Implementation of the ABCDEF bundle compared with previous studies before and after the COVID-19 pandemic.

Element of the ABCDEF bundle	This study		Reference^a^	Reference^b^	Reference^c^
Before or after the pandemic	After the COVID-19 pandemic			Before the COVID-19 pandemic	
Survey date	27th January in 2021		3rd June and 1st July in 2020	January 2015–March 2017	March–September in 2016
Survey settings	International		International	National (USA)	International
Target ICU patients	WITHOUT COVID-19 infection	WITH COVID-19 infection	WITH COVID-19 infection	WITHOUT COVID-19 infection	
Number of enrolled patients	(*n* = 627)	(*n* = 602)	(*n* = 262)	(*n* = 15,226)	(*n* = 1,521)
An entire of the ABCDED bundle	(0%)	(1%)	(1%)	(8%)	
Element A, *n* (%)	(64%)	(55%)	(45%)	(77%)	(83%)
Element B (both SAT and SBT)	(17%)	(10%)			
Spontaneous awakening trial, *n* (%)	(21%)	(14%)	(28%)	(34%)	(66%)
Spontaneous breathing trial, *n* (%)	(29%)	(16%)	(28%)	(36%)	(67%)
Element C, *n* (%)	(45%)	(61%)	(52%)	(59%)	(89%)
Element D, *n* (%)	(39%)	(35%)	(39%)	(56%)	(70%)
Element E, *n* (%)	(12%)	(16%)	(35%)	(29%)	
Element F, *n* (%)	(16%)	(30%)	(16%)	(63%)	(67%)

*Data presented as number (%). SAT, spontaneous awakening trial; SBT, spontaneous breathing trial*.

a *Liu et al. (12)*.

b *Pun et al. (13)*.

c *Morandi et al. (21)*.

Differences in the implementation of elements of the ABCDEF bundle might be caused by differences in underlying diseases and ICU length of stay between the two groups. Patients with COVID-19 infections, admitted to the ICU because of severe respiratory failure (28), need longer mechanical ventilation treatment with more sedation and require more intense sedation monitoring compared to patients without COVID-19 infections who have a variety of reasons for admission to the ICU but result in a shorter ICU length of stay. This might lead to more frequent implementation of element C for patients with COVID-19 infections. However, the deeply sedated state could result in non-implementation of element A as seen in patients with COVID-19 infections because few pain assessment tools can be used in heavily sedated patients. The strong respiratory drive and effort associated with COVID-19 infections could be a factor contributing to progression of lung injury (29–31) and could prevent conduct of SAT and SBT for patients with COVID-19 infection, to avoid further exacerbation of the lung injury. However, element F of the ABCDEF bundle was more frequently performed for patients with COVID-19 infections, especially using electronic devices (using a monitor such as phone/video). COVID-19 brought technological expansion in the fields of remote and tele-practice which have been applied to the COVID-19 situation (32). However, it might result in overlooking patients without COVID-19 infections who received less online benefits as shown in this survey, and also need involvement of their families. In the context of an increasing trend for implementation of element E (33, 34), COVID-19 brought it to a previous level, but into more resource-unbalanced and time-restricted settings. As the guideline suggests, it is important to note that evidence-based ICU care, such as the ABCDEF bundle and ICU diary, should be incorporated into clinical practice for all ICU patients regardless of their underlying diseases or the ICU length of stay (1–4, 16, 35).

After adjusting for the backgrounds of hospitals and ICUs and the baseline condition of patients, the presence of COVID-19 infection was not a barrier to the implementation of each element of the ABCDEF bundle. For elements D, E, and F, the presence of COVID-19 was significantly associated with their implementation. The warnings of high risk for and high incidence of delirium in the early stage of the pandemic may be a factor (36) or the use of online systems in patients with COVID-19 infections (32) might contribute to these results. In addition, this could be a strong message that the impact of the COVID-19 pandemic broadly affected patients without and with COVID-19 infections and special considerations are necessary to improve the quality of ICU care for both types of patients.

This study demonstrates the diversity of independent factors associated with the implementation of each element of the ABCDEF bundle in addition to variations comparing non-COVID-19 and COVID-19 settings. These results particularly show that a promising strategy to introduce or implement a specific element of the bundle in an ICU could vary and should be designed depending on the context and local situation in which it will be implemented. For many elements in the ABCDEF bundle, regardless of COVID-19 status, a specific protocol and presence of a target/goal for ICU care were consistently identified as facilitating independent factors. However, this study also showed the low frequency to equip the specific protocol in each ICU, or 50% or less, which could be considered as one of the major barriers to be managed regardless of the presence of COVID-19. As many studies successfully showed a pivotal role for implementation or introduction of ICU care, this simple, but not time- or resource-consuming approach could be a key stimulus and should be routine in the ICU to facilitate efficient implementation of evidence-based approaches to ICU care (12, 16, 37, 38). Tele-medicine, which is getting public interest and recommended in several elements such as elements E (39) and F (37, 40), could be also an alternative to promote implementation instead of strict regulations regarding infection control or family visits. This is a relatively novel field of intensive care. Therefore, the impact of tele-medicine on implementation of evidence-based ICU care and its effect on outcomes should be investigated in a large prospective cohort study or randomized controlled study. The professionals dedicated to the ICU and the individual with primary responsibility could be decided by a policy maker in the hospital or ICU director based on what is to be achieved (41–44). The income level, used as a resource barometer, might show that less resources prevent implementation of evidence-based approaches (17, 37, 45). Relatively resource-intense care, such as a 1:1 nurse-patient ratio and daily multidisciplinary rounds, were not independently associated with implementation of the ABCDEF bundle, consistent with a previous report (12). ICUs in lower income countries performed more element F in this survey. These countries might apply relatively flexible visiting hours, which was also detected as a facilitating factor for element F, for a family rather than being in a high-income country.

This study has several acknowledged limitations. First, the limited number of patients and participating countries (Japan accounts for 40%) could lead to selection bias and limit generalizability to other ICUs and countries. These numbers might not be enough for the multivariable analysis with a number of covariates. Although the survey date captured a peak in the wave in Japan (46), the status of the pandemic in each area could affect the results. In addition, some COVID-19 hotspots, such as the USA, Brazil, and Russia, were under-represented. Second, the nature of a point prevalence study does not define a causal relationship and reflects the overwhelming situation at participating sites. This point prevalence study took place entirely on 1 day. Third, potential confounding factors associated with implementation, such as disease-related factors, were not investigated. Finally, an odds ratio with a relatively broad confidence interval may indicate an unstable model created by multivariate analysis. For example, Tele-ICU availability and Visiting hours might not be suitable to be incorporated into the multivariable analysis. Interpreting the results into the clinical world needs cautions regarding these statistical aspects. Further investigation and observations are necessary to validate these results.

## Conclusions

Though having a COVID-19 infection was not associated with a failure to implement evidence-based ICU care, the implementation rates for the entire ABCDEF bundle, each of its elements and the ICU diary for patients without and with COVID-19 infections, were various, but extremely low on the whole regardless of the presence of COVID-19 infection. Since the impact of the COVID-19 pandemic on evidence-based ICU care varies depending on the conditions in each ICU, strategies to facilitate the implementation of each element of the ABCDEF bundle must be tailored to each institution.

## Data Availability Statement

The raw data supporting the conclusions of this article will be made available by the authors, without undue reservation.

## Ethics Statement

The studies involving human participants were reviewed and approved by the Ethics Committee of the Saiseikai Utsunomiya Hospital. Written informed consent from the participants' legal guardian/next of kin was not required to participate in this study in accordance with the national legislation and the institutional requirements.

## Author Contributions

KL, KN, HK, PN, EE, SK, KT, SI, and ON: study conception and design. KL, KN, HK, PN, EE, SK, and KT: statistical analysis or interpretation of data and drafting the manuscript. KN, HK, ME, PN, EE, SK, KT, MG, BL, CC, JB, SI, AL, and ON: critical review and revision of the manuscript for important intellectual insight. PN, EE, SK, KT, SI, AL, and ON: study supervision. KL, KN, HK, ME, PN, EE, SK, MG, BL, CC, JB, SI, and ON: recruitment the participating ICUs in overseas countries. KN confirmed that all authors meet authorship criteria according to ICMJE. All authors drafted the manuscript for important intellectual content, contributed to revision of the final version of the manuscript, approved the final version submitted, and agreed to be accountable for all aspects of the work in ensuring that questions related to the accuracy or integrity of any part of the work are appropriately investigated and resolved.

## Conflict of Interest

KL reports personal fees from MERA and receives a salary from TXP Medical completely outside the submitted work. KN reports personal fees from Abbott Laboratory, Nestle, TERUMO, GETINGE, Asahi Kasei Pharma, Ono Pharmaceutical, Japan Blood Products Organization, Nihon Pharmaceutical, Otsuka Pharmaceutical, Pfizer, Toray, and Baxter, and grants from Asahi Kasei Pharma outside the submitted work. HK receives a salary from the Japanese Society for Early Mobilization (non-profit society) as a chair (full time) outside the submitted work. EE reports grants from the VA/NIH; personal fees from Pfizer, Orion, and Lilly; personal fees from Masimo; and grants from Kohler outside the submitted work. SI reports personal fees from MERA, Abbott Laboratory, Teijin Pharma, Nestle, and Nihon Pharmaceutical. ON reports grants from Asahi Kasei Pharma, Ono Pharmaceutical, Baxter, Maruishi Pharmaceutical, Torii Pharmaceutical, Teijin Pharma, Shionogi Pharmaceutical, and Fuso Pharmaceutical outside the submitted work. The remaining authors declare that the research was conducted in the absence of any commercial or financial relationships that could be construed as a potential conflict of interest.

## Publisher's Note

All claims expressed in this article are solely those of the authors and do not necessarily represent those of their affiliated organizations, or those of the publisher, the editors and the reviewers. Any product that may be evaluated in this article, or claim that may be made by its manufacturer, is not guaranteed or endorsed by the publisher.
