# Supplementary material for: Implementation of the ABCDEF Bundle for Critically Ill ICU Patients During the COVID-19 Pandemic: A Multi-National 1-Day Point Prevalence Study
Source: Front Med (Lausanne). 2021 Oct 28;8:735860. doi: 10.3389/fmed.2021.735860 (PMC8581178; doi:10.3389/fmed.2021.735860)
Supplement: Supplementary file 1 [file Table_1.docx]

Supplementary Material

Supplemental Table 1. Additional information on background, structure and policies of participating hospitals and ICUs

Data in table are presented as number (%). ICU intensive care unit. **(a)** This includes 8 physicians other than Intensivists (6%) and respiratory therapists 1(1%)

| **Characteristic** | **Participating ICUs (n=135)** |
| --- | --- |
| Type of hospital, n (%) |  |
| University hospital | 56 (41%) |
| University affiliated hospital | 35 (26%) |
| Community hospital | 35 (26%) |
| Others | 9 (7%) |
| Type of ICU, n (%) |  |
| Medical-Surgical mixed ICU | 103 (76%) |
| Medical ICU | 14 (10%) |
| Surgical ICU including cardiac surgery | 13 (10%) |
| Pediatric ICU | 2 (1%) |
| Other | 3 (2%) |
| Professionals dedicated to the ICU, n (%) |  |
| Intensivist | 123 (91%) |
| Physiotherapist | 72 (53%) |
| Occupational therapist | 15 (11%) |
| Respiratory therapist | 36 (27%) |
| Nutritionist dietitian | 51 (38%) |
| Pharmacist | 67 (50%) |
| Primary responsibility to make decisions on implementing the ABCDEF bundle in the ICU, n (%) |  |
| Multidisciplinary/-professional rounds / conference / team | 39 (29%) |
| Nurse | 9 (7%) |
| Intensivist | 53 (39%) |
| Other ^a^ | 9 (7%) |
| No introduction of the ABCDEF bundle in the ICU | 23 (17%) |
