# Supplementary material for: Implementation of the ABCDEF Bundle for Critically Ill ICU Patients During the COVID-19 Pandemic: A Multi-National 1-Day Point Prevalence Study
Source: Front Med (Lausanne). 2021 Oct 28;8:735860. doi: 10.3389/fmed.2021.735860 (PMC8581178; doi:10.3389/fmed.2021.735860)
Supplement: Supplementary file 2 [file Table_2.docx]

Supplementary Material

Supplemental Table 2. Details of factors independently associated with implementation of each element of the ABCDEF bundle

Data are presented as odds ratios [95% Confidence Interval] (*: p value <0.05, **: <0.01, ***:<0.001). ICU intensive care unit, SAT spontaneous awakening trials, SBT spontaneous breathing trials.

| **Element** | ***A*** | **B: Spontaneous Awakening Trials** | **B: Spontaneous Breathing Trials** | **C** | **D** | **E** | **F** |
| --- | --- | --- | --- | --- | --- | --- | --- |
| **COVID-19 infection positive** | 0.68  [0.41-1.10] | 0.80  [0.38-1.67] | 1.59  [0.81-3.14] | 1.42  [0.80-2.52] | **3.04*****  [1.86-4.97] | **2.97****  [1.48-5.96] | **3.15*****  [1.60-6.20] |
| **Presence of a specific written protocol** | **1.77****  [1.16-2.70] | **4.22*****  [2.52-7.06] | 1.59  [0.97-2.60] | **1.82****  [1.18-2.81] | 1.12  [0.80-1.57] | 1.68  [0.98-2.89] | 1.08  [0.63-1.86] |
| **Application of a target or goal to ICU patients** | **6.46**^***^  [4.53-9.22] |  |  | **6.87*****  [4.56-10.4] |  | **10.6*****  [6.09-18.4] |  |
| **Number of ICU beds** | 1.01  [1.00-1.03] | 0.99  [0.97-1.02] | 1.01  [0.98-1.03] | 1.01  [0.99-1.03] | 0.98  [0.97-1.00] | 1.01  [0.99-1.04] | 0.98  [0.96-1.00] |
| **Number of ICU beds allocated to patients with COVID-19 infections** | 1.01  [1.00-1.03] | 1.00  [0.98-1.03] | 0.99  [0.96-1.01] | 1.02  [1.00-1.04] | **1.02***  [1.01-1.04] | **0.97****  [0.95-1.00] | 0.99  [0.97-1.00] |
| **Tele-ICU Availability** | **4.76**^**^  [1.72-13.10] | **36.5*****  [9.47-141] | **29.3*****  [8.65-99.1] | **4.81****  [1.62-14.3] | **12.2*****  [4.73-31.5] | 1.30  [0.39-4.32] | **9.62**^***^  [3.31-27.90] |
| **Nurse: patient ratio: 2** | ***REFERENCE*** | | | | | | |
| **1** | **0.44**^***^  [0.27-0.71] | **0.37****  [0.18-0.79] | **0.45***  [0.23-0.91] | **0.28*****  [0.16-0.51] | **0.37*****  [0.22-0.62] | 1.51  [0.77-3.00] | 0.76  [0.41-1.41] |
| **>3** | **0.55**^*^  [0.31-0.98] | 0.69  [0.29-1.66] | 0.84  [0.38-1.90] | **0.13*****  [0.07-0.26] | **0.46***  [0.26-0.84] | 0.64  [0.29-1.41] | 0.79  [0.39-1.60] |
| **Frequency of multidisciplinary rounds: Not applicable** | ***REFERENCE*** | | | | | | |
| **Daily** | 1.05  [0.68-1.62] | 1.31  [0.68-2.53] | 1.08  [0.59-2.00] | 1.21  [0.74-1.99] | **2.28*****  [1.48-3.53] | 1.23  [0.66-2.30] | 1.10  [0.63-1.90] |
| **Other (at least weekly or monthly)** | 1.56  [0.77-3.19] | 0.64  [0.22-1.89] | 2.10  [0.85-5.18] | 0.95  [0.43-2.12] | **3.17*****  [1.59-6.29] | 1.44  [0.52-3.99] | 1.17  [0.44-3.16] |
| **Visiting hours: NONE** | ***REFERENCE*** | | | | | | |
| **0< x ≤6** | 1.25  [0.86-1.83] | 1.02  [0.58-1.77] | 1.35  [0.81-2.23] | 0.76  [0.49-1.18] | 1.35  [0.92-1.98] | **2.01***  [1.16-3.48] | 2.58  [1.51-4.40] |
| **6≤ x ≤24** | **25.4*****  [6.70-95.80] | 1.35  [0.29-6.44] | 0.96  [0.25-3.69] | 2.63  [0.96-7.21] | 0.45  [0.18-1.12] | 0.30  [0.07-1.37] | 10.80  [3.03-38.70] |
| **Professionals dedicated to the ICU: Intensivist** | **3.94*****  [2.07-7.50] | **0.29***  [0.13-0.68] | **0.25*****  [0.12-0.54] | **3.33****  [1.62-6.82] | 0.94  [0.51-1.74] | 0.76  [0.29-1.97] | **5.89****  [1.98-17.50] |
| **Physiotherapist** | **1.92***  [1.16-3.18] | 0.91  [0.47-1.76] | 1.40  [0.77-2.57] | **0.55***  [0.32-0.95] | **0.34*****  [0.22-0.55] | 0.68  [0.34-1.37] | **0.45***  [0.24-0.85] |
| **Occupational therapist** | 1.12  [0.69-2.15] | **0.20****  [0.07-0.56] | 0.49  [0.22-1.11] | **2.90****  [1.53-5.51] | 0.76  [0.43-1.37] | 1.32  [0.62-2.81] | 1.61  [0.78-3.35] |
| **Respiratory therapist** | 078  [0.49-1.24] | 0.70  [0.37-1.33] | 0.80  [0.46-1.38] | 0.79  [0.48-1.32] | **1.90****  [1.25-2.88] | 1.31  [0.73-2.36] | **2.10****  [1.24-3.55] |
| **Nutritionist** | **0.52***  [0.31-0.89] | **3.31*****  [1.65-6.64] | 1.14  [0.59-2.18] | 0.85  [0.48-1.49] | 0.80  [0.49-1.30] | 1.26  [0.62-2.59] | **6.05*****  [3.14-11.71] |
| **Pharmacist** | 0.99  [0.68-1.44] | 0.66  [0.37-1.16] | 1.19  [0.73-1.96] | 0.73  [0.47-1.15] | 1.07  [0.73-1.56] | 0.63  [0.35-1.11] | **0.33*****  [0.20-0.56] |
| **Primary responsibility for the ABCDEF bundle: None / others** | ***REFERENCE*** | | | | | | |
| **Multidisciplinary team** | **1.57***  [1.05-2.35] | **2.02***  [1.03-3.93] | **2.72****  [1.47-5.06] | 0.71  [0.44-1.14] | **0.55****  [0.36-0.83] | 1.37  [0.70-2.66] | 1.40  [0.78-2.50] |
| **Intensivist** | 0.71  [0.41-1.22] | **3.45****  [1.45-8.21] | **3.06****  [1.37-6.83] | 0.97  [0.51-1.84] | 1.28  [0.74-2.20] | 1.31  [0.55-3.13] | 1.33  [0.60-2.98] |
| **Nurse** | 1.02  [0.39-2.68] | 2.23  [0.71-6.99] | **6.27*****  [2.20-17.90] | 0.94  [0.32-2.77] | **2.35***  [1.01-5.46] | 3.19  [0.97-10.50] | 0.12  [0.01-1.58] |
| **Income level: High income** | ***REFERENCE*** | | | | | | |
| **Low- and lower middle-income** | **0.39*****  [0.24-0.63] | 1.08  [0.53-2.2] | 1.71  [0.86-3.38] | **0.17*****  [0.10-0.29] | **0.18*****  [0.11-0.29] | 0.87  [0.41-1.83] | 1.25  [0.67-2.32] |
| **Upper middle-income** | 0.62  [0.35-1.10] | 1.05  [0.42-2.64] | **3.18****  [1.43-7.08] | 1.32  [0.65-2.70] | **0.50***  [0.26-0.95] | 0.81  [0.34-1.89] | **6.02*****  [3.03-12.00] |
