# Supplementary material for: Implementation of the ABCDEF Bundle for Critically Ill ICU Patients During the COVID-19 Pandemic: A Multi-National 1-Day Point Prevalence Study
Source: Front Med (Lausanne). 2021 Oct 28;8:735860. doi: 10.3389/fmed.2021.735860 (PMC8581178; doi:10.3389/fmed.2021.735860)
Supplement: Supplementary file 3 [file Table_3.docx]

Supplementary Material

Supplemental Table 3. Details of differences in independently associated factors in patients without and with COVID-19 infections

Data are presented as odds ratios [95% Confidence Interval] (*: p value <0.05, **: <0.01, ***:<0.001). ICU intensive care unit, SAT spontaneous awakening trials, SBT spontaneous breathing trials. Blank cells are variables excluded from multivariable logistic regression analysis because the number of patients in the category is too small (≤5 patients) to create a suitable model or the variables were removed through the stepwise method with Akaike information criterion.

| **Element** | ***A*** | | **B: Spontaneous Awakening Trials** | | **B: Spontaneous Breathing Trials** | | **C** | | **D** | | **E** | | **F** | |
| --- | --- | --- | --- | --- | --- | --- | --- | --- | --- | --- | --- | --- | --- | --- |
| **Factor** | ***Non- COVID*** | **COVID** | ***Non-COVID*** | **COVID** | ***Non-COVID*** | **COVID** | ***Non-COVID*** | **COVID** | ***Non-COVID*** | **COVID** | ***Non-COVID*** | **COVID** | ***Non-COVID*** | **COVID** |
| **Presence of a specific written protocol** | **2.37**^***^  [1.45-3.87] | **2.43**^**^  [1.31-4.52] | **4.25**^***^  [2.46-7.34] | **5.84**^***^  [2.49-13.70] |  | **3.36**^***^  [1.69-6.67] | **2.10**^*^  [1.15-3.81] |  |  | **1.83***  [1.01-3.32] |  |  |  | **37.6**^***^  [13.8-102.00] |
| **Application of a target or goal to ICU patients** | **5.31**^***^  [3.33-8.46] | **11.20**^***^  [6.08-20.50] |  |  |  |  | **6.61**^***^  [3.95-11.10] | **19.4**^***^  [9.00-41.90] |  |  | **10.7**^***^  [5.20-22.20] | **14.4**^***^  [6.33-32.80] |  |  |
| **Number of ICU beds** | **1.02**^*^  [1.00-1.40] |  | **0.94**^***^  [0.91-0.97] |  |  | 1.02  [0.99-1.04] | 1.02  [0.99-1.05] |  | **0.96**^**^  [0.94-0.99] | **0.97**^**^  [0.96-0.99] |  | **1.03**^*^  [1.00-1.05] |  |  |
| **Number of ICU beds allocated to patients with COVID-19 infections** |  | **1.03**^***^  [1.02-1.05] |  |  | **0.94**^*^  [0.89-0.99] | 0.98  [0.96-1.00] |  | **1.03**^***^  [1.02-1.05] |  | **1.03**^***^  [1.01-1.05] | **0.91**^**^  [0.86-0.97] | 0.98  [0.95-1.00] |  |  |
| **Tele-ICU Availability** |  |  | **14.30**^**^  [2.19-93.70] | **30.00**^***^  [6.71-134.00] |  | **28.50**^***^  [8.60-94.40] | **11.80**^***^  [2.89-47.90] |  | **32.2^***^**  [6.41-161.00] |  |  | **3.63***  [1.24-10.60] | **7.77**^**^  [2.07-29.20] |  |
| **Nurse patient ratio: 2** | ***REFERENCE*** | | | | | | | | | | | | | |
| **1** | 0.62  [0.33-1.15] | **0.26****  [0.11-0.60] |  |  |  |  | 0.79  [0.41-1.52] | **0.35**^*^  [0.14-0.87] |  | **0.34**^**^  [0.17-0.71] |  | 1.71  [0.84-3.47] |  |  |
| **>3** | **0.34**^**^  [0.16-0.72] | 0.49  [0.17-1.37] |  |  |  |  | **0.23**^**^  [0.09-0.63] | **0.15**^***^  [0.05-0.42] |  | **0.21**^***^  [0.09-0.51] |  | 0.54  [0.20-1.47] |  |  |
| **Frequency of multidisciplinary rounds: Not applicable** | ***REFERENCE*** | | | | | | | | | | | | | |
| **Daily** |  |  |  | 1.20  [0.49-2.97] | 0.46  [0.18-1.15] | 0.62  [0.29-1.33] |  | 0.24  [0.09-0.68] | 1.25  [0.58-2.71] | 0.47  [0.22-1.00] |  | 0.54  [0.20-1.47] |  | 1.88  [0.84-4.24] |
| **Other (at least weekly or monthly)** |  |  |  | **0.16**^**^  [0.04-0.57] | 0.92  [0.34-2.47] | **0.31^*^**  [0.12-0.83] |  | **0.32**^*^  [0.10-0.96] | **2.39***  [1.02-5.62] | 1.40  [0.64-3.07] |  |  |  | **10.70**^***^  [4.57-24.90] |
| **Visiting hours:**  **NONE** | ***REFERENCE*** | | | | | | | | | | | | | |
| **0< x ≤6** | 1.25  [0.76-2.04] | **0.27*****  [0.13-0.56] |  |  |  |  |  |  |  | **0.26**^***^  [0.14-0.48] |  |  | **3.84**^***^  [1.78-8.92] | **1.90***  [1.12-3.21] |
| **6≤ x ≤24** | **10.00**^***^  [2.78-36.30] |  |  |  |  |  |  |  |  |  |  |  | **15.00**^***^  [4.89-46.20] |  |
| **Professionals dedicated to the ICU: Intensivist** | 1.75  [0.83-3.67] | **31.7*****  [9.34-107.00] |  | **0.16**^**^  [0.05-0.48] | **0.29**^**^  [0.11-0.72] | **0.36**^*^  [0.13-0.99] | 1.95  [0.84-4.55] | **19.10**^***^  [4.87-75.10] | **3.08**^**^  [1.48-6.41] | **0.12**^***^  [0.04-0.35] |  |  |  |  |
| **Physiotherapist** |  | 2.16  [0.94-4.96] |  |  | 1.57  [0.84-2.91] |  |  |  |  | **0.32**^***^  [0.17-0.57] |  |  | **0.35**^*^  [0.15-0.84] |  |
| **Occupational therapist** | **3.30**^**^  [1.39-7.81] |  | **0.16**^**^  [0.04-0.62] | **0.28**^*^  [0.09-0.86] |  | **0.27**^**^  [0.10-0.72] |  | **6.56**^***^  [2.66-16.20] | **0.13**^***^  [0.04-0.39] |  |  |  |  |  |
| **Respiratory therapist** |  |  |  | **0.29**^**^  [0.11-0.72] |  | **0.37**^*^  [0.17-0.82] | **2.18^*^**  [1.12-4.23] |  | **2.35**^*^  [1.18-4.66] | **2.87**^***^  [1.62-5.08] |  | **2.60**^**^  [1.3-5.18] | **5.84**^***^  [2.99-11.40] |  |
| **Nutritionist** | **0.45**^**^  [0.29-0.76] | **0.21**^***^  [0.09-0.52] | **1.90**^*^  [1.05-3.41] | **3.20**^**^  [1.36-7.50] |  | 2.18  [0.92-5.14] | **0.30**^***^  [0.16-0.57] | **0.41**^*^  [0.18-0.97] | **0.48**^**^  [0.28-0.83] |  | **2.31**^*^  [1.07-4.98] |  | **6.03**^***^  [2.86-12.70] | 1.67  [0.95-2.92] |
| **Pharmacist** |  |  | **0.51**^*^  [0.29-0.90] |  |  |  |  |  |  | 0.67  [0.39-1.14] | **0.44**^**^  [0.24-0.80] |  | **0.34**^***^  [0.17-0.65] |  |
| **Primarily responsibility of the ABCDEF bundle: None / others** | ***REFERENCE*** | | | | | | | | | | | | | |
| **Multidisciplinary team** | **2.53**^**^  [1.34-4.76] |  |  |  | 1.69  [0.63-4.59] |  | **0.23**^**^  [0.16-0.66] | **0.35**^*^  [0.15-0.82] | **0.43**^**^  [0.23-0.81] |  | **4.24**^**^  [1.6-11.20] |  |  |  |
| **Intensivist** | 0.96  [0.54-1.72] |  |  |  | **2.95**^*^  [1.17-7.39] |  | 0.55  [0.29-1.06] | 0.85  [0.36-2.00] | 0.61  [0.34-1.12] |  | **3.74**^**^  [1.51-9.25] |  |  |  |
| **Nurse** | 0.95  [0.38-2.36] |  |  |  | **8.59**^***^  [2.72-27.10] |  | 0.52  [0.18-1.51] | 0.89  [0.22-3.60] | 2.09  [0.89-4.94] |  | **11.20**^***^  [3.50-36.10] |  |  |  |
| **Income level: High income** | ***REFERENCE*** | | | | | | | | | | | | | |
| **Low and lower middle income** | **0.17**^***^  [0.09-0.32] | 0.52  [0.23-1.14] |  |  | 1.18  [0.41-3.38] |  | **0.23**^***^  [0.11-0.49] | **0.18**^***^  [0.08-0.43] | **0.11**^***^  [0.05-0.23] | **0.36**^***^  [0.20-0.63] |  |  | **2.27**^*^  [1.00-5.15] | 1.38  [0.74-2.59] |
| **Upper middle income** | 0.71  [0.37-1.35] | **0.23**^**^  [0.09-0.62] |  |  | **8.25**^***^  [3.22-21.10] |  | 1.33  [0.50-3.52] | 0.55  [0.18-1.71] | **0.21**^***^  [0.09-0.46] | **0.41***  [0.18-0.95] |  |  | **6.18**^***^  [2.89-13.20] | **7.24**^***^  [3.58-14.60] |
