# Supplementary material for: Implementation of the ABCDEF Bundle for Critically Ill ICU Patients During the COVID-19 Pandemic: A Multi-National 1-Day Point Prevalence Study
Source: Front Med (Lausanne). 2021 Oct 28;8:735860. doi: 10.3389/fmed.2021.735860 (PMC8581178; doi:10.3389/fmed.2021.735860)
Supplement: Supplementary file 4 [file Data_Sheet_1.docx]

Supplementary Material

Appendix 1: List of Collaborators and Site Investigators

**NATIONAL SOCIETIES / NETWORKS WHICH ENDORSED THIS STUDY:**

Indian Society of Critical Care Medicine (ISCCM): President of the Indian Society of Critical Care Medicine, Dr. Dhruva Chaudhry; Korean Society of Critical Care Medicine (KSCCM): President of the Korean Society of Critical Care Medicine, Dr. Sang Hyun Kwak; ICU Recovery Network; Japanese Society of Early Mobilization (JSEM); Japanese Society of Education for Physicians and Trainees in Intensive Care (JSEPTIC); Infectious Diseases Association for Teaching and Education in Nippon (IDATEN); Emergency Medicine Alliance (EMA);

Social Networking Service: Facebook, Twitter

Web-site (https://forms.gle/sUJfLDpYoJ9nHDZr7)

**NATIONAL COORDINATORS OUTSIDE OF JAPAN**

The role of national coordinators is to recruit hospitals in their local area through their own personal networks or local societies to which they belong.

| Region | Country | Name | Institution |
| --- | --- | --- | --- |
| Europe | Germany | **Peter Nydahl, RN** | Nursing Research, Department of Anesthesiology and Intensive Care Medicine, University Hospital of Schleswig-Holstein, Kiel, Germany. |
| Africa | Libya | **Muhammed Elhadi, MBBCh** | Faculty of Medicine, University of Tripoli, Tripoli, Libya |
| Asia | India | **Mohan Gurjar, M.D.** | Department of Critical Care Medicine, Sanjay Gandhi Post Graduate Institute of Medical Sciences (SGPGIMS), India |
| Asia | Malaysia | **Be Kim Leong, M.D., MRehabMed** | Department of Rehabilitation Medicine, Sarawak General Hospital, Kuching, Sarawak |
| Asia | Korea | **Chi Ryang Chung, M.D., Ph.D.** | Department of Critical Care Medicine, Samsung Medical Center, Sungkyunkwan University School of Medicine, Seoul, Korea |
| Asia | Singapore | **Balachandran Jayachandran, M.Phty** | Rehabilitation Department, Woodlands Health Campus, Singapore |

**ISIIC Ⅱ COMMITTEE INFORMATION**

Office address: Level 2, 1-2-12, Kudankita, Chiyoda-ku, Tokyo, 102-0073

Office email: [isiic2@jsea2005.org](about:blank)

Office TEL: +81-3-3556-5585

Staff: Tomoya Kuroda

**ISIIC Ⅱ STEERING COMMITTEE MEMBERS**

Keibun Liu, Kensuke Nakamura, Hajime Katsukawa, Muhammed Elhadi, Peter Nydahl, Eugene Wesley Ely, Sapna R. Kudchadkar, Shigeaki Inoue, Osamu Nishida

**ONLINE QUESTIONNAIRE CREATION-**

**DATA SECURITY, REVISION AND MANAGEMENT:**

Tomohiro Sonoo: Hitachi General Hospital, Hitachi, Ibaraki, TXP Medical Co. Ltd., Tokyo), Takumi Ochiai: TXP Medical Co. Ltd., Tokyo

**SITE INVESTIGATORS**

One representative from each ICU was registered. If there are names of several representatives in one hospital, it means that there are different ICUs registered, with different functions and locations or the principal representative needs other investigators’ help because of the overwhelming situation during the COVID-19 pandemic.

| Country | Hospital | Representatives |
| --- | --- | --- |
| **ALBANIA** | University Hospital Center " Mother Theresa ", Tirana | Ilir Ohri  Dariel Thereska |
| **ALGERIA** | Ibn Sina Hospital | Kouidri Khadidja |
| **ANDORRA** | Hospital Nostra Senyora de Meritxell | Antoni Margarit Ribas |
| **ARGENTINA** | Hospital Italiano de Buenos Aires  Centro Gallego de Buenos Aires  Luis Lagomaggiore | Nicolas A. Gemelli  Luis Alejandro Boccalatte  Graciela Zakalik |
| **AUSTRALIA** | The Royal Children's Hospital Melbourne | Shinya Miura |
| **BANGLADESH** | Ibn Sina Medical College Hospital  United Hospital  Asgar Ali Hospital, Dhaka | Tarikul Hamid  Mohammed Salah Uddin  Md Motiul Islam |
| **BRAZIL** | Hospital municipal ruth cardoso  Hospital Memorial Arthur Ramos  Hospital Carvalho Beltrão  Complexo Hospitalar Regional Deputado Janduhy Carneiro | Pedro Salomão  Igor Lima Buarque  Gustavo Mendonça Ataíde Gomes  Rebeca Dias Rodrigues Araújo |
| **COLOMBIA** | Hospital San Vicente Fundación Medellín | Edward Blandón |
| **CYPRUS** | Near East University Hospital | Heyam Almezghwi |
| **EGYPT** | Assiut University Hospital  Prince Sultan Hospital  Minia University Hospitals  Sohag general hospital  Aswan University Hospital  Berkit el sabaa general hospital  Alexandria Main University Hospital  Kafr Elsheikh University hospital  Tanta University Hospital  El Safa Hospital  Ain-Shams university  National Cancer Institute, Cairo University  Berkit el sabaa general hospital  Elmabara hospital  Benha University Hospital | Aliae AR Mohamed Hussein  Aliaa Abd Rabo  Nehal Gamal Omar  Ahmed Mohammed Abu-Elfatth  Ibrahim Fawzy ELgouhary Abdelfattah  Yasmin K. NasrEldin  Monica Dobs  Islam Galal  Mohamed Hellmy Zaki Zagho  Hanan M. Hemead  Sarah M. Hemead  Eslam M. Khalaf  Mostafa Mahmoud Tayeb  Mohammad Elbahnasawy  Muhammad Hamad  Ahmed Mahmoud Mohamed Saad  Wafaa Abdelsalam  Mohamed Elbahnasawy  Ahmed Y Azzam  Ahmed K. Awad  Galal Ghaly  Mohamed Hellmy Zaki Darwish Zagho  Neama mashhout  Ahmed Elshafey  Ahmed Abdelmoein  Ahmed Abdelsadek |
| **EL SALVADOR** | General Hospital. Salvadoran Social Security Institute. | Carlos E. Orellana-Jimenez |
| **FRANCE** | Hôpital Nord Franche-Comté  Groupe Hospitalier Sud Ile-de-France, Hôpital de Melun-Sénart | Fernando Daniel Berdaguer Ferrari  Sebastien Jochmans |
| **GREECE** | Athens General Hospital of Evaggelismos  Saint Paul ("Agios Pavlos") General Hospital  University Hospital of Ioannina | Irini Patsaki  Theodoros Aslanidis  Georgios Papathanakos |
| **GUATEMALA** | Hospital Roosevelt | Zonia Guzman |
| **INDIA** | Sir Gangaram Hospital  Tagore Hospitel JALANDHR  Sanjay Gandhi Postgraduate Institute of Medical Sciences (SGPGIMS)  Virinchi Hospital  Apollo hospitals  Royalcare superspeciality hospital  Health City Hospital  BKL Walawalkar Hospital, Chiplun  VPMH Hospital, Pune  Santosh Medical College Hospital .Ghajiabad  Believers Church Medical College Hospital  Manipal Hospital  Yashoda Hospital, Somajiguda, Hyderabad,  AIIMS, Patna  AIIMS Rishikesh | Niraj Tyagi  dr ziyokov joshi  Mohan Gurjar  Srinivas Samavedam  Saroj Kumar Pattnaik  Lakshmikanthcharan  Chandana Sarma  Amol Hartalkar  Sheetal Hartalkar  Anil Kumar  Sanjo Sunny  Gautham M Raju  Kaladhar S  Divendu Bhushan  Nidhi Gupta |
| **IRAN** | Imam Reza Hospital | Ata Mahmoodpoor |
| **IRAQ** | Al-Hilla Teaching Hospital  Arzheen Private Hospital  Babylon Maternal and Children  Zafaraniyah General Hospital  Alhakim General Hospital in Najaf | Ali Al-Isawi  Hayder Yousif Alhasan  Haiderbareh  Rand Hussein  Maytham Al-Juaifari |
| **ITALY** | Sant'Andrea Hospital  Ospedale "Magalini" - Villafranca di Verona | Monica Rocco  Plinio Calligaro |
| **JORDAN** | Irbid Speciality Hospital  Jordanian Royal Medical Services- King Hussein Medical Center  Islamic Hospital | Almu'atasim Khamees  Amro Mohammad Abuleil    Bourhan Alrayes |
| **JAPAN** | Teine Keijinkai hospital  Kobe University Hospital  Kagoshima City Hospital  Nagasaki University Hospital  Sapporo City General Hospital  Tokushima University Hospital  Ageo Central General Hospital  Tokushima Prefectural Central Hospital  Shinshu University Hospital  Okayama Saiseikai General Hospital  Niigata University Hospital  Fukuyama City Hospital  Okinawa Kyodo Hospital  Japanese Red Cross Kyoto Daiichi Hospital  Urasoe General Hospital  Mie University Hospital  Hyogo Prefectural Amagasaki General Medical Center  Saga University Hospital  Okayama University Hospital  Fukuoka University Hospital  Chugoku Rosai Hospital  Shinkomonji Hospital  International University of Health and Welfare Narita Hospital  Wakayama Medical University  St. Marianna University, School of Medicine, Yokohama-city Seibu Hospital  University of Tokyo Hospital  Minaminagano Medical Center Shinonoi General Hospital  Hirosaki University Hospital  Saiseikai Utsunomiya Hospital  Osaka General Medical Center  National Hospital Organization Tokyo Medical Center  Yokohama Municipal Citizen's Hospital  National Hospital Organization Disaster Medical Center  Obihiro-Kosei Hospital  Nishijima Hospital  Hamamatsu University Hospital  Otsu City Hospital  Hitachi General Hospital  Tokyo Medical and Dental University  Japanese Red Cross Maebashi Hospital  University of Tsukuba Hospital  Kasugai Municipal Hospital  Jikei University Kashiwa Hospital  Nagoya Medical Center  National Defense Medical College Hospital  Sakai City Medical Center  Nara Prefecture General Medical Center  University Hospital Kyoto Prefectural University of Medicine  Fujisawa City Hosipital  Showa University Hospital  Sendai City Hospital  Tokyo Metropolitan Tama Medical Center  Mito Saiseikai General Hospital  Hiroshima University Hospital  Toyooka Public Hospital Tajima Emergency and Critical Care Medical Center  National Hospital Organization Kyoto Medical Center  Tohoku University Hospital  Japanese Red Cross Medical Center  Sakakibara Heart Institute  Naha City Hospital  Tsuchiura Kyodo General Hospital  Yokohama City Minato Red Cross Hospital  Tokyo Medical University Ibaraki Medical Center  Hyogo Emergency Medical Center  Fujita Health University  Takatsuki general hospital  Kanazawa University Hospital  Kagawa University  Yokosuka Kyosai Hospital  SUBARU Health Insurance Society Ota Memorial Hospital | Takako Akimoto  Moritoki Egi  Masataka Nakamura  Tetsuya Hara  Masahiro Takahashi  Nobuto Nakanishi  Fumiko Kambe  Yuta Arai  Hiroshi Kamijo  Takashi　Hongo  Masakazu Nitta  Kenzo Ishii  Yutaka Sakuda  Masahito Horiguchi  Munekatsu Miyahira  Tadashi Kaneko  Masaru Matsumoto  Ayaka Matsuoka  Hiromichi Naito, Hiroshi Morimatsu  Yuhei Irie  Tatsutoshi Shimatani  Naoki Tominaga  Kazuya Omura  Kyohei Miyamoto  Akiyoshi Nagatomi  Naoki Hayase  So Oishi  Shinya Yaguchi  Tetsuro Kamo  Takeshi Nishida  Junji Hatakeyama  Hajime Hayami  Kazushige Inoue  Mamoru Komatsu  Kazuma Watanabe  Satoshi Asai  Toshihiko Yokotani  Kensuke Nakamura  Yuka Mishima  Hiroyuki Suzuki  Yuki Enomoto  Toshimichi Takahashi  Tatsuhiko Abe  Yasunari Morita  Kohei Yamada  Junko Kimura  Tomoya Yamaguchi  Ayako Noguchi  Osamu Akasaka  Fumihito Kasai  Yoshinobu Kameyama  Jun Hamaguchi  Yoshiki Tamatsukuri  Kohei Ota  Daisuke Taniguchi  Satoru Beppu  Takuya Shiga  Fumiaki Ishikawa  Takumi Nagao  Daisetsu Yasumura  Kenji Oike  Kei Sugiki  Kunio Yanagita  Shinichi Ijuin  Tomoyuki Nakamura  Chihiro Takayama  Masaki Okajima  Tomonori Sugawara  Yutaka Usuda  Kazuki Akieda |
| **KOREA, REP.** | Inje University Busan Pail Hospital  Samsung Medical Center  Korea University Anam Hospital  Gyeongsang National University Changwon Hospital  Armed Forces Capital hospital  Eunpyeong St. Mary's Hospital  Wonkwang University Hospital | Sukyoon Lee  Chi Ryang Chung  Jae-Seung Jung  Ho Cheol Kim  Donghoon Kim  Sei Won Kim  Chul Park |
| **LEBANON** | Nini Hospital | Ibrahim Salah el Din |
| **LIBYA** | Tripoli Central Hospital  Aljalla hospital  Sabha medical center  El Khadra Hospital  Nephro center  Ibn Sina  Althowra hospital  Oncology hospital musrata  Military field isolation hospital / Ajdabiya  Tripoli Medical Hospital  Raiaina hospital  Martyr Attia alkaseh teaching hospital  Almogarif hospital  Alkhadra hospital  Benghazi Medical Center  Alshahid Attia Alkasah General Hospital  Tobruk medical center  Tripoli children hospital  Dar Alshifa Hospital  Alhadba alkhadra hospital  Brega General Hospital  Elmarj teaching hospital  Paediatric Benghazi Hospital  Elmarj teatching hospital  Zliten Medical Center  Misurata Central Hospital  Diabetes and endocrine hospital  Chest center  Airport road polyclinic  Nalut central hospital  Sorman Teaching Hospital  Mietiga hospital  Misurata Medical Center  Sebha Medical Center  Almwasfat Isolation Center | Eman Abdulwahed  Duha Milad Abdullah  Ibrahim Ellojli  Abdussalam mady  Khaled aboumreeqa  Ahmed Buimsaedah  Safa mohammed Alfadheel  Aiman Ali Salem  Elham Braieg  Safia Adam Mosa  Mohammed Abdelkabir  Sana Moussa Shagour  Salmin Ibrahim Matoug  Shoukrie I. Shoukrie  Rafiq Boozed  Almoatasemalzanaty  Wesam ebrahim  Aihab ben amoor  Hana M Al-Gataani  Sulayman Almabrouk Sulayman Meelad  Ghadah alarbish  Wejdan Ali Alhadi  Mohammed YAHYA  Fatimah majeed Ali  Aml Ahmed Egbeta  Abdaljalel-A-Alzwai  Hana alfaytouri Alwaer alkeelani  TAHA ABUBAKER  Adel Gessel  Mohammed Abdalraheem Huwaysh  Khalid M.G Mohammed  Surour Salem Almabrouk  Abdulmuez Abdulmalik  Malek Mohamed Abusannuga  Almajdoub Ali Mohammed Ali  Emad Amkhatirah  Mabroukah saeid alshamikh  Yousef ibrahim hamad  Salma Muftah Omran  Abobaker Elbarouni  Fatimah Mohammed Bin Alsagheer  Hasan Almusrati  Rema husien  Mona Masaud Amro  Abdulmueti Alhadi  Abdulkarim Aldoukali Babaa  Abdalmageed alsharif alghenai  Saedah Abdeewi  Abdurraouf abusalama |
| **MALAYSIA** | International Islamic University Malaysia Medical Centre  University of Malaya Medical Centre  Sarawak General Hospital (Paediatric ICU)  Sarawak General Hospital (Neurosurgery ICU)  Sarawak General Hospital (Adult General ICU-Covid Ward)  Sarawak General Hospital (Adult General ICU-Non Covid Ward)  Hospital Raja Permaisuri Bainun | Mohd Basri MAT-NOR  Nor'azim Mohd Yunos  Huong Nai Law  Richard Teo Soon Kiat  Wan Daud Wan Kadir  Be Kim Leong  Shivani Rajasegaran |
| **MOROCCO** | Hassan 2 hospital  Zouneir skirj  Haut Grand Atlas  Mohammed V Military Hospital Rabat  Military Hospital Moulay Ismail  Ibn sina rabat  Hôpital Al Farabi ( ex Hopital maurice losteau) | Ayoub Ait lahcen  Salma ait lachgar  A.e.assia elhachmi  Ait Bouachrine Sarah  Balkhi Hicham  Yousra Zouine  Khalifa omar  Mohammed Leknani |
| **NAMBIA** | Windhoek Central Hospital | Ndatiyaroo W. Agapitus |
| **NEPAL** | Nepal Mediciti Hospital | Rashmi Suvedi |
| **NETHERLANDS** | Gelre Hospitals  Franciscus Gasthuis en Vlietland | Marleen Flim  Victor van Bochove |
| **NIGERIA** | Abubakar Tafawa Balewa University Teaching Hospital | Musa Abubakar Madaki |
| **OMEN** | Khoula Hospital | John Massoud |
| **PALESTINE** | Al Shifa Hospital  Alia Govermental Hospital  Rafidiah Hospital | Muawia S.J. Alkhazendar  Ghassan Al-Saikaly  Mustafa Abu Jayyab  Sarah Amro  Othman Mustafa |
| **PHILIPPINES** | Asian Hospital and Medical Center | Joanne Robles |
| **POLAND** | University Clinical Center , Gdansk | Tomasz Zwolinski |
| **PORTUGAL** | Centro Hospitalar Vila Nova de Gaia/Espinho  Centro hospitalar universitário São João | Ana Rios  Ana Afonso |
| **QATAR** | Hamad General Hospital | Ahmed S. Humadi Alsheikhly |
| **ROMANIA** | Fundeni Clinical Institute | Dana R Tomescu |
| **RUSSIA** | FSBI «NATIONAL MEDICAL RESEARCH CENTER FOR OBSTETRICS, GYNECOLOGY AND PERINATOLOGY NAMED AFTER ACADEMICIAN V.I.KULAKOV» MINISTRY OF HEALTHCARE OF THE RUSSIAN FEDERATION | Alexey Pyregov |
| **SAUDI ARABIA** | Prince Sultan Miltary Medical City  Prince Sultan military hospital Taif region  KING SAUD HOSPITAL | Ghaleb A.Almekhlafi  Ibrahim fawzy ELgouhary Abdelfattah  Osama Sobh |
| **SINGAPORE** | Singapore General Hospital  Woodlands Health Campus  Khoo Teck Puat Hospital | Vimal Palanichamy  Jayachandran Balachandran  Tan Boon Chai Sunny |
| **SOUTH AFRICA** | Groote Schurr Hospital | Ranem Sherif |
| **SPAIN** | Vall Hebron Institute of Research  Hospital Universitario de Getafe  Hospital Clínic Universitari de València  Hospital Verge de la Cinta de Tortosa, Tarragona  Hospital Clínico Universitario Lozano Blesa (Zaragoza)  Hospital Francesc de Borja Gandia | No name available  Fernando Frutos-Vivar  Rafael Badenes  Ferran Roche-Campo  Herrero García, Sandra  Susana Isabel Gil Garcia |
| **SUDAN** | Al Hakiem hospital  Al-Mak Nimir University Hospital  khartoum Isolation Center  Yastabshiroon Hospital  Wad Medani Teaching Hospital | Amani Ibrahim Abakar Bargo  Ahmed Osama Ahmed Babikir  Nusaiba Hassan Mohamed Eltahir  Shahd Elsiddig Ali Suliman  Mahmoud Saleh |
| **SWIZERLAND** | Spital Bülach  Clinique Cecil,Hirslanden | Bernd Yuen  Fleisch Isabelle |
| **SYRIA** | Al-Mouwasat University Hospital  Sham hospital  Tishreen University Hospital  Islamic Hospital Amman | Mohammad Karam Chaaban  Ahmad Mahmoud Hmaideh  Alaa Hamdan  Mohammed Sultan Amaereh |
| **TURKEY** | Karadeniz Technical University and Trabzon Kanuni Hospital Zonuldak Atatürk State Hospital  Düzce University Hospital | Ahmet Eroglu  Mahmud Islam  Türkay Akbaş |
| **UNITED ARAB EMIRATES** | Prime Hospital | Dirar Abdallah |
| **UNITED KINGDOM** | Our Lady of Lourdes Hospital  Queen Elizabeth the Queen Mother Margate  Gateshead Foundation Trust  University Hospitals Coventry and Warwickshire NHS Trust  Blackpool Teaching Hospitals NHS Trust  Queen Elizabeth Hospital Birmingham  Medway Maritime Hospital | Tharwat Aisa  Tarek Metwally  Hatim Albirnawi  David McWilliams  Nicky Williams  Jonathan Weblin  Sarah Elliott |
| **UNITED STATES** | Keck Medical Center of USC  UC Davis Health  Comer Children's Hospital, University of Chicago  Interfaith Medical Center  University of California San Diego  Lincoln Medical Center  Vassar Brothers Medical Center | John Margetis  Sarina A. Fazio  Neelima Marupudi  Ramakanth Pata  Robert L. Owens  Mohammad Aldiabat  Mazin Shaikhoun, MD |
| **VENEZUELA** | Hospital Central "Dr. Miguel Pérez Carreño" | Ingrid T. von der Osten R |
| **YEMEN** | Zaid hospital Sana'a city | Rafat Ameen Mohammed Al-saban |
|  |  |  |
