# Supplementary material for: Implementation of the ABCDEF Bundle for Critically Ill ICU Patients During the COVID-19 Pandemic: A Multi-National 1-Day Point Prevalence Study
Source: Front Med (Lausanne). 2021 Oct 28;8:735860. doi: 10.3389/fmed.2021.735860 (PMC8581178; doi:10.3389/fmed.2021.735860)
Supplement: Supplementary file 5 [file Data_Sheet_2.docx]

Supplementary Material

Appendix 2. Hospital and ICU background questions

**Survey of basic information of the hospital/ICU**

Dear Study Participants,

The aim of this study is to describe the implementation of daily ICU care, especially associated with the ABCDEF bundle, PADIS guidelines, and nutrition for all ICU patients regardless of COVID-19 infection status.

We appreciate your interest in participating in this online survey. Please look over the information below carefully before agreeing to participate by clicking **‘Agree to participate’** at the bottom.

1. This investigation consists of two surveys:

Survey of basic information of the hospital/ICU (17 questions, 3-5 minutes to complete)

Survey of evidence-based and supportive ICU care (21 questions, 3-5 minutes to complete per ICU patient).

1. **Do NOT forget** to save the **Facility Registration Number** you will be given when you complete this survey of basic information of the hospital/ICU. You will need to enter this number when you answer the survey for daily ICU care on 27 January 2021. If you forget the number, you need to complete this survey again to get a new Facility Registration Number. The Study Committee does not save and cannot give you the number to protect anonymity.
2. You will be given about 20 questions associated with the ICU care you are providing to patients on 27 January 2021 in the survey of daily ICU care. The questionnaire includes information about age, gender, and estimated Body Mass Index, which are collected as categorial variables. You need to complete one questionnaire about ICU care for each patient. It should take about 3 to 5 minutes per questionnaire.
3. Your participation in this survey is voluntary. If you decide to join the study and start to answer, you may withdraw at any point during the questionnaire for any reason before submitting your answers by pressing the ‘Submit’ button/closing the browser.
4. This project has been reviewed by, and received ethical approval from, the Saiseikai Utsunomiya Hospital Institutional Review Board in Japan [2020-69] as central institutions of this study. The ISIIC Study Committee is investigating ethical issues in collaboration with a lawyer in Japan. We believe that ethical review from each facility is not required according to ethical policies in Japan. This study is conducted via a survey that does not include personal information as defined in the Personal Information Protection Law in Japan and is therefore judged to meet the requirements for ethics review exemption in the ethical guidelines for medical research as described below.
5. The collected information does not include data that can be used to identify the facility or individual.
6. We do not use samples taken from the human body.
7. This is an observational study that does not involve any intervention or any burden on personnel.
8. Registered institutions definitely have the right to decide whether they choose to answer the questions.
9. The content of the questions will not cause psychological distress to the respondents. Respondents in registered facilities are guaranteed the right to refuse to answer the questionnaire and are not disadvantaged or coerced into answering the questionnaire if they do not return it.
10. This study is aiming to include participating sites mainly in Asia, Europe, and Africa. We may exclude data from the United States and Oceania because of regional issues. Even if your data is excluded from the primary analysis, the name of the representative will be included in the Acknowledgement.

However, please note that each institution outside of Japan must consider the necessity for ethical review in their institution.

**The name of one representative from each participating facility will be included as an acknowledgement in all study publications.**

If you have any questions or concerns about any aspect of this survey, please do not hesitate to contact to the ISIIC 2 study committee below. We will do our best to answer your query and resolve it.

**ISIIC 2 Study Committee Mail: isiic2@jsea2005.org**

The principal investigators of this study are: Keibun Liu M.D., Ph.D., Kensuke Nakamura M.D., Ph.D., Hajime Katsukawa PT, Ph.D., and Osamu Nishida, M.D., Ph.D.

If you agree to participate after reading the above, please click **“Agree to participate”** at the bottom of this web page to start the questionnaire. If you do not agree to participate in this survey, please click **“Disagree to participate”** at the bottom to close this web page.

- **Agree to participate**
- **Disagree to participate**

**For all questions, please select the one best answer unless otherwise indicated.**

**Responder identity**

1. What is your role in the ICU?

- Nurse (include nurse managers, directors, and critical care nurse specialists)
- Intensivist (Physician)
- Physician other than intensivist
- Dedicated Physiotherapist in the ICU
- Non-dedicated physiotherapist
- Respiratory therapist
- Other

**Hospital Characteristics**

1. Which country are you working in?
2. How many beds does your hospital have?

- n<200 beds
- 200≦ n＜400
- 400≦ n＜600
- 600≦ n＜800
- n≧800

1. Describe the academic affiliation of your hospital.

- University hospital
- University-Affiliated hospital
- Community hospital
- Others

**ICU Structure / Characteristics**

1. What is the type of ICU?

- Medical
- Medical-surgical (mixed)
- Surgical
- Cardiac surgical
- Neurologic
- Pediatric
- Others

1. Is your ICU managed as a tele-ICU by another hospital or ICU?

- Yes
- No

1. How many ICU beds does your ICU have?
2. How many ICU beds are **specifically designated for patients with COVID-19**?
3. What is the nurse-to-patient ratio in your ICU?

- 1
- 2
- 3
- 4
- ≧5

1. Are these professionals dedicated to your ICU? (Click all that apply)

- Nurses
- Intensivist
- Physiotherapist
- Occupational therapist
- Respiratory therapist
- Nutritionist / dietitian
- Pharmacist
- None

1. What is the number of visiting hours in your ICU for a family per day in following situations?

(1) ★ For a family before the COVID-19 pandemic. ★

- No visiting hours available
- 0< n <6 hours
- 6≦ n＜12
- 12≦n ＜18
- 18≦ n＜24
- No limitation on visiting hours

(2) ★ For a family of a patient WITHOUT COVID-19, after the COVID-19 ★pandemic started

- No visiting hours available
- 0< n <6 hours
- 6≦n ＜12
- 12≦n ＜18
- 18≦n ＜24
- No limitation on visiting hours

(3) ★　For a family of a patient WITH COVID-19 infection ★

- No visiting hours available
- 0< n<6 hours
- 6≦ n＜12
- 12≦ n＜18
- 18≦ n＜24
- No limitation on visiting hours

1. Who may enter the room of patients with COVID-19 infections under the infection control regulations of your hospital? (Click all that apply)

- Nurses (include nurse managers, directors, and critical care nurse specialists)
- Intensivists
- Physicians other than intensivists
- Dedicated Physiotherapist in the ICU
- Non-dedicated physiotherapists
- Occupational therapists
- Respiratory therapists
- Facility management, e.g. for cleaning
- No limitations / no regulations

1. How many times daily does your ICU have multi-professional/-disciplinary rounds to visit patients WITH COVID-19 infection?

- Not applicable
- At least once daily
- At least once a week
- once a month
- other

1. How many times daily does your ICU have multi-professional/-disciplinary rounds to visit patients WITHOUT COVID-19 infection?

- Not applicable
- At least once daily
- at least once a week
- once a month
- other

1. Are there written protocols shown below in your ICU? (Click all that apply)

- Pain management protocol (assess, prevent and manage Pain)
- Spontaneous Awakening Trial (SAT) management protocol
- Spontaneous breathing trial (SBT) management protocol
- Sedation management protocol
- Delirium management protocol (assess, prevent and manage Delirium)
- Early mobility and exercise protocol
- Family engagement and empowerment protocol
- Nutrition management protocol
- Physical restraint protocol
- ICU Diaries protocol
- No protocol
- Other

16. Who is primarily responsible for implementing the ABCDEF bundle in your ICU? If you have a primarily responsibility, it means that you take a key role to make decision on how the bundle will be provided to the patient.

- Multidisciplinary/-professional rounds / conference / team
- Nurse (include nurse managers, directors, and critical care nurse specialists)
- Intensivist (Physician)
- Physician (other than an intensivist)
- Physiotherapist (dedicated to the ICU)
- Physiotherapist (not dedicated to the ICU)
- Respiratory therapist
- No one has responsibility for implementing the bundle
- The ABCDEF bundle is not implemented in the ICU
- Others

17. What kinds of scale for ICU care do you use in your ICU? (Click all that apply)

- - Numerical Rating Scale (NRS)
  - Critical-care Pain Observation Tool (CPOT)
  - Behavioral Pain Scale (BPS)
  - Escala de Conductas Indicadoras de Dolor (ESCID)
  - Visual Analogue Scale for Pain assessment
  - Richmond Agitation- Sedation Scale (RASS)
  - Sedation-Agitation Scale (SAS)
  - Ramsay Sedation Scale
  - Confusion Assessment Method for ICU (CAM-ICU)
  - Intensive Care Delirium Screening Checklist (ICDSC)
  - Others
  - None

Caution！！

**Do NOT forget** to save the **Facility Registration Number** you will be given when you complete this survey of basic information of the hospital/ICU. You will need to enter this number when you answer the survey for daily ICU care on 27 January 2021.

If you forget the number, you need to complete this questionnaire again to receive another Facility Registration Number.

18. Is this questionnaire you just completed a repeat entry (for example if you lost the Facility Registry Number)?

- Yes
- No
