# Supplementary material for: Implementation of the ABCDEF Bundle for Critically Ill ICU Patients During the COVID-19 Pandemic: A Multi-National 1-Day Point Prevalence Study
Source: Front Med (Lausanne). 2021 Oct 28;8:735860. doi: 10.3389/fmed.2021.735860 (PMC8581178; doi:10.3389/fmed.2021.735860)
Supplement: Supplementary file 6 [file Data_Sheet_3.docx]

Supplementary Material

Appendix 3: Survey of evidence-based and supportive ICU care

- **You need to answer for all ICU patients.**
- **You need to complete one questionnaire (for ICU care) for each patient. For example, if you have three patients in your ICU, you need to complete three questionnaires.**
- **When you want to go to a questionnaire for the next patient, you must complete the current questionnaire first, then click “Next daily ICU care” or re-open the URL to answer for the next patient.**
- **Your colleagues can help by using the Facility Registration Number simultaneously to answer the survey about daily ICU care for other patients. For example, nurse A answers about ICU care for patient B while doctor C answers about ICU care for patient D at the same time with the same Facility Registration Number.**

**For all questions, please select the one best answer unless otherwise indicated.**

**You do not need to answer these questions for patients receiving terminal or end of life care.**

**Facility Registration Number**

1. Please write the **Facility Registration Number** you received after completing the hospital/ICU information questionnaire. If you forgot it, please complete the survey for basic information of hospital/ICU again and get a **new facility registration number**.

(survey of basic information is at URL: <https://forms.gle/aM7xdUHqZUiVNEDP8>　 )

**Daily ICU care provided to this patient**

1. Is this patient admitted to your ICU because of COVID-19 infection? COVID-19 is defined as a disease caused by a SARS-CoV-2 infection which is laboratory confirmed by RT-PCR.

Yes

No

1. How many days has this patient been in the ICU? (days)
2. What is the age of this patient (x)?

- x<20 years old
- 20≦x＜30
- 30≦x＜40
- 40≦x＜50
- 50≦x＜60
- 60≦x＜70
- 70≦x＜80
- x≧80

1. What is the patient’s gender?

- Male
- Female
- Other

1. What is the estimated Body Mass Index of your patient (x)?

- x<18.5
- 18.5≦x＜25
- 25≦x＜30
- 30≦x＜35
- x≧35

The questionnaire of daily ICU care starts here. Please answer this questionnaire based specifically on the daily ICU care you are providing or provided for this patient today, 27 January 2021.

1. What kind of respiratory assistance did the patient receive today? (click all that apply)

- No respiratory assistance
- Oxygen, such as nasal cannula, face mask, reserved face mask, and others
- Nasal high flow cannula
- Non-invasive ventilation
- Mechanical ventilation
- Veno-Venous Extracorporeal membrane oxygenation
- Veno-Arterial Extracorporeal membrane oxygenation
- Other

1. Choose the treatment you are giving to the patient today (click all that apply)

- Continuous/Intermittent renal replacement therapy
- Intra-Aortic Balloon Pumping (IABP)
- Impella^®^
- Continuous use of neuromuscular blockage
- Continuous use of vasoactive drugs
- Continuous use of analgesia agents
- Continuous use of sedation agents
- Other
- None of the above

1. What is the total number of hours of ‘prone positioning’ you provided for this patient today.

- Not applicable (e.g., because of no respiratory failure)
- 0 hours
- 0< x<6 hours
- 6≦x＜12
- 12≦x＜18
- 18≦x＜24
- 24 hours

1. What sedatives do you give continuously to this patient? (click all that apply)

- No sedative agents used
- Benzodiazepine
- Propofol
- Dexmedetomidine
- Barbiturate
- Inhaled sedation
- Remifentanil
- Other

**Daily ICU care associated with the ABCDEF bundle and other supportive care**

1. Did the patient receive following ICU care associated with the ABCDEF bundle today? (click all that apply)

- Regular standardized PAIN assessment using valid and reliable pain assessment scales (※１) 6 times or more per day.
- Spontaneous Awakening Trial (SAT) assessment (※２)
- Spontaneous Breathing Trial (SBT) assessment (※３)
  - Regular standardized SEDATION assessment using valid and reliable sedation assessment scales (※4) 6 times or more per a day.
- Regular standardized delirium assessment using valid and reliable delirium monitoring tools (※5) 2 times or more per day.
- Mobility activities that were out of bed or higher (※6) (It is equal to a score of 4 or higher according to the Intensive Care Unit Mobility Scale shown in Question 12)
- Family member/significant other of this patient is educated on the ABCDEF bundle and/or participate in at least one of the followings: rounds; conference; plan of care; or ABCDEF bundle related care, e.g., re-orientation, calming talks etc.
- The education and participation of family member/significant was conducted online.
- ICU diary (※7)
- Physical restraints on the bed at any time for the patient today

※１ The pain assessment scales include Numerical Rating Scale (NRS), Critical-care Pain Observation Tool (CPOT), Behavioral Pain Scale (BPS), and others.

※２ SAT is cessation of sedatives and narcotics or similar protocol to evaluate consciousness

※３ SBT is to turn the respiratory rate to zero with 8 or less of pressure support ventilation or similar local protocol to evaluate whether the patient meets the requirements for extubation

※４ The sedation assessment scales include Richmond Agitation- Sedation Scale (RASS), Sedation-Agitation Scale (SAS), Ramsay Sedation Scale, and others.

※５ The delirium assessment tools include Confusion Assessment Method for ICU (CAM-ICU), Intensive Care Delirium Screening Checklist (ICDSC), and others.

※６ i.e., dangling at edge of bed, standing at side of bed, walking to bedside chair, marching in place, walking in room or hall.

※7 An ICU diary is a patient journal, written by staff and families for several purposes, and includes daily entries about what happened.

1.
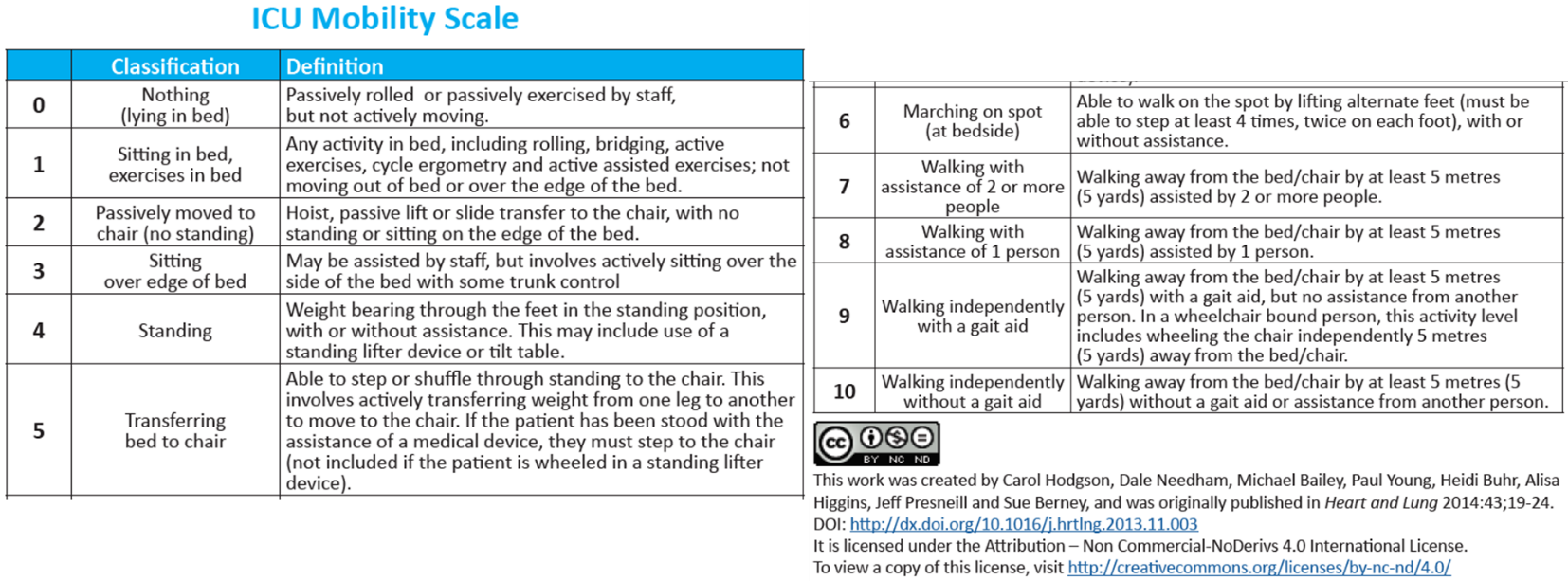
What was the highest mobility level of the patient today according to the Intensive Care Unit Mobility Scale? (See below- Intensive Care Unit Mobility Scale)
2. For days with limited mobility, select why she/he did NOT achieve the mobility level of sitting over edge of bed or more which is equal to a score of 4 or higher according to the Intensive Care Unit Mobility Scale. (Choose the most important barriers to rehabilitation)

- The Intensive Care Unit Mobility Scale was a score of 4 or higher
- Consciousness factor (existing consciousness disorder, RASS: ≤ -3 or ≥+2, deep sedation, delirium, etc.)
- Subjective symptoms (respiratory distress, BPS or > 3 or NRS > 5, fatigue, patient refusal, etc.)
- Respiratory factor (SpO2: <90%; FIO2: >0.6; respiratory rate: >30 times/min、ventilator unsynchronized, etc.)
- Circulatory factor (systolic blood pressure: <90 or >180 mmHg; mean blood pressure: <65 or >110 mmHg; heart rate: <50 or >120 beats/min; new arrhythmias; additional administration of vasopressors, etc.)
- Device factor (exist catheter, drain, dialysis, mechanical ventilation, or extracorporeal membrane oxygenation, etc.)
- Medical staff factor (lack of staff, holidays, many examinations, poor time adjustment, etc.)
- Factors associated with COVID-19 (restriction for medical staff to contact with the patients, restriction for rehabilitation, infectious control, etc.)
- Others

1. Do you have a target or goal on following today’s ICU care? (Click all that apply)

- Pain (i.e. targeted number on the pain scale)
- Sedation (i.e. targeted number on the sedation scale)
- Mobilization / Rehabilitation (i.e. targeted rehabilitation level)
- Nutrition (i.e. targeted energy or protein provision)
- Other
- No target or goal on ICU care

1. Was the patient diagnosed with delirium by the assessment tool, such as Confusion Assessment Method for ICU (CAM-ICU), Intensive Care Delirium Screening Checklist (ICDSC), and others? (put yes if positive on either assessment that day)

- No tool used for delirium assessment
- Yes
- No
- Not applicable (i.e. because of no consciousness)

1. Do you provide the patient with non-pharmacological interventions to control delirium today? (click all that apply)

- None
- Physical environment intervention (changing light application, earplugs, use of a mirror, acoustic or visual stimulation, restraint use avoidance)
- Orientation strategy
- Sedation reducing (spontaneous awaking trials, stop use of benzodiazepine or narcotics, etc)
- Family participation (orientation in family’s voice, nurse‐facilitated family participation in psychological care, )
- Exercise program (introduction of standardized rehabilitation protocol, strengthen mobilization/rehabilitation duration, frequency, or intensity, etc)
- Cerebral hemodynamic improving (doppler and oximetry monitoring, angioplasty to optimize cerebral blood flow, optimize mean arterial blood pressure or oxygenation, etc)
- Support for senses (hearing aids/glasses)
- Maximize sleep condition / standardized sleep protocol
- Sunbathing
- Multi-component program (implementation of ABCDEF bundle, combination of two or more interventions above)
- Other interventions

1. What mobility device/devices were used for this patient today? (click all that apply)

- No device
- Portable ergometer on the bed
- Neuro Muscular Electrical Stimulation
- Lift up device, ceiling lifter
- Tilt belt
- Walker
- Hip and Ankle Linked Orthosis (HALO)
- Others

1. Could the family meet with this patient today or see the patient using a monitor such as a phone and video today? (click all that apply)

- No
- In person
- Visiting through the glass outside the room
- Using an electronic device (using a monitor such as phone / video)

1. How do you provide nutrition for the patient? (click all that apply)

- Total parenteral nutrition
- Enteral nutrition (non-oral)
- Oral
- No nutrition

1. Total energy (kcal) of nutrition (x) provided within the last 24 hours (from yesterday until this morning) (In case of oral nutrition, please estimate based on the actual amount of intake)

- ＜10 (kcal/kg)
- 10≦x＜20 (kcal/kg)
- 20≦x＜30 (kcal/kg)
- x≧30 (kcal/kg)

1. Total protein (g/kg) provided within the last 24 hours (from yesterday until this morning) (In case of oral nutrition, please estimate based on the actual amount of intake)

- ＜1.2g/kg
- ≧1.2g/kg

We greatly appreciate your help and kindness.

You can complete the information of daily ICU care you provided to this patient by clicking “Submit” below. If you want to complete this survey for another patient with COVID-19, please click ‘Submit another response’ in the next page.

**Submit**

**(Next page)**

- If you want to complete this survey for another patient with COVID-19, please click ‘Submit another response’
- If you want to end the questionnaire or re-start later, please close this page.
